# Alginate Inhibits Iron Absorption from Ferrous Gluconate in a Randomized Controlled Trial and Reduces Iron Uptake into Caco-2 Cells

**DOI:** 10.1371/journal.pone.0112144

**Published:** 2014-11-12

**Authors:** Anna A. Wawer, Linda J. Harvey, Jack R. Dainty, Natalia Perez-Moral, Paul Sharp, Susan J. Fairweather-Tait

**Affiliations:** 1 Norwich Medical School, University of East Anglia, Norwich, Norfolk, United Kingdom; 2 Institute of Food Research, Norwich, Norfolk, United Kingdom; 3 King's College London, London, United Kingdom; University of Florida, United States of America

## Abstract

Previous *in vitro* results indicated that alginate beads might be a useful vehicle for food iron fortification. A human study was undertaken to test the hypothesis that alginate enhances iron absorption. A randomised, single blinded, cross-over trial was carried out in which iron absorption was measured from serum iron appearance after a test meal. Overnight-fasted volunteers (n = 15) were given a test meal of 200 g cola-flavoured jelly plus 21 mg iron as ferrous gluconate, either in alginate beads mixed into the jelly or in a capsule. Iron absorption was lower from the alginate beads than from ferrous gluconate (8.5% and 12.6% respectively, p = 0.003). Sub-group B (n = 9) consumed the test meals together with 600 mg calcium to determine whether alginate modified the inhibitory effect of calcium. Calcium reduced iron absorption from ferrous gluconate by 51%, from 11.5% to 5.6% (p = 0.014), and from alginate beads by 37%, from 8.3% to 5.2% (p = 0.009). *In vitro* studies using Caco-2 cells were designed to explore the reasons for the difference between the previous *in vitro* findings and the human study; confirmed the inhibitory effect of alginate. Beads similar to those used in the human study were subjected to simulated gastrointestinal digestion, with and without cola jelly, and the digestate applied to Caco-2 cells. Both alginate and cola jelly significantly reduced iron uptake into the cells, by 34% (p = 0.009) and 35% (p = 0.003) respectively. The combination of cola jelly and calcium produced a very low ferritin response, 16.5% (p<0.001) of that observed with ferrous gluconate alone. The results of these studies demonstrate that alginate beads are not a useful delivery system for soluble salts of iron for the purpose of food fortification.

**Trial Registration:**

ClinicalTrials.gov NCT01528644

## Introduction

Iron fortification is a widely used strategy to reduce the risk of iron deficiency but it presents a major challenge to the food industry. Water soluble forms of iron, such as ferrous gluconate [1], are generally more bioavailable than non-soluble iron compounds, but they can cause adverse organoleptic changes when added to foods [2]. Iron chelators such as ascorbic acid, sodium ethylenediaminetetraacetic acid (EDTA) and nicotianamine increase iron bioavailability by preventing the formation of insoluble ferric hydroxide and protecting the iron from binding with substances such as phytate which render the iron unavailable for absorption [3], [4], [5]. However, the complexes may be unstable during food processing and storage, and/or cause discoloration of the food products [5], [6]. Alginates bind divalent metals and could provide an alternative delivery system to maximise iron absorption in iron fortification programmes.

Alginates are natural copolymers present in the cell walls of brown seaweed as sodium, potassium, calcium and magnesium salts of alginic acid. They are comprised of varying ratios of two different acids: D-mannuronic and L-guluronic acid, and due to the variable length of the polymer chains they exhibit differing physiochemical properties [7]. Alginates are widely used in variety of applications, including the food industry (as thickening, gelling, emulsifying and stabilising agents in food products (ice cream, sauces, fruit pies) [8] and drug delivery systems (anti-reflux preparations) [9]. They have been shown to bind divalent and trivalent cations and form a stable complex with iron (III) over a range of different pH values [10], [11], and therefore may provide a useful vehicle for soluble iron compounds used to fortify foods.

Previous in vitro studies demonstrated that alginate solutions had an enhancing effect on iron uptake (measured by ferritin formation) in a Caco-2 cell model system, and alginate beads containing ferrous gluconate delivered more available iron than beads containing ferric ammonium citrate [12]. In contrast, studies in ileostomy patients suggested a potential inhibitory effect of alginate on iron absorption; but as the study was underpowered no clear conclusions could be drawn [13].

The aim of this work was to examine the effect of alginate on iron absorption in order to determine whether it could be a useful mechanism for fortifying selected foods with iron. Iron absorption from alginate beads saturated with ferrous gluconate and ferrous gluconate on its own were compared in a group of human volunteers. In addition, the effect of calcium on iron absorption was investigated. Calcium is required for alginate bead production but is a known inhibitor of iron absorption in single meal studies [14]. The first hypothesis tested was that alginate in iron-containing alginate beads conferred protection to the iron in the gastrointestinal tract and delivered more available iron for absorption into the mucosal cells of the duodenum than ferrous gluconate administered in a gelatine capsule. The second hypothesis tested was that calcium in the gut lumen would bind to alginate thereby reducing its inhibitory effect on iron absorption. Following on from the human study, *in vitro* experiments were performed to identify possible explanations for the *in vivo* findings.

## Materials and Methods

### Human study

**Figure 1:**
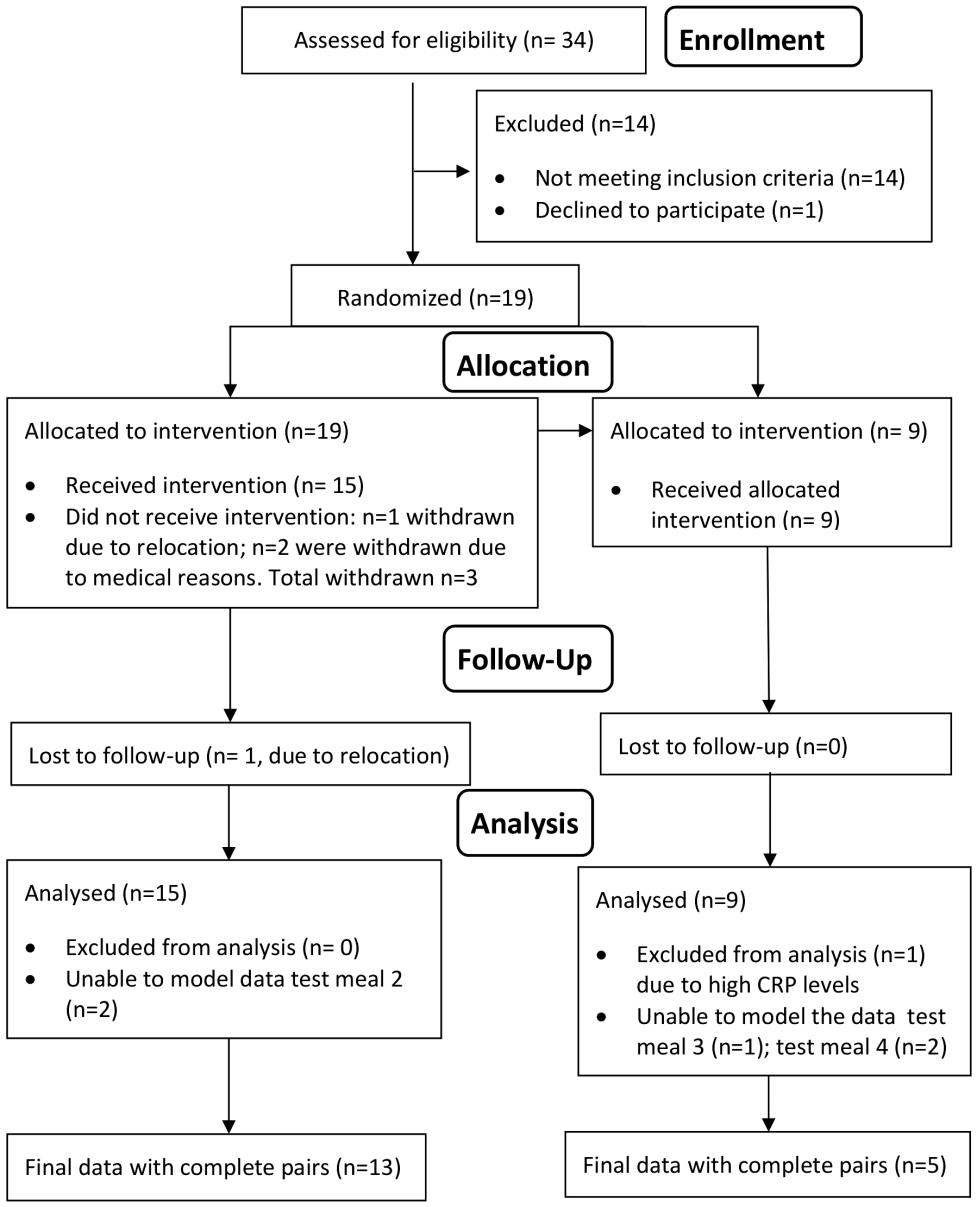
Flow diagram of human study. Number of participants at every stage (CONSORT statement).

Volunteers were selected from populations with a higher prevalence of low iron stores, namely women of child-bearing age (18–45 years old) and men who were regular blood donors (aged 18–65 years old). Iron absorption is up-regulated when ferritin values are low to moderate (ferritin <60 µg/L) [14], thus selecting individuals with a ferritin <60 µg/L increases the sensitivity of the absorption test. Volunteers were recruited locally from advertisements, websites and email. Recruitment started on 12^th^ March 2012 and finished on 3^rd^ August 2012. All appointments (initial interview, screening visit and study days) were carried out by the study scientist and took place in the Clinical Research Facility at the University of East Anglia Norwich Medical School (Figure 1). The study participants were young females aged 19–27 y, with the exception of one male, aged 49 y. Subject characteristics are presented in Table 1 and individual data in Table S1. Each person underwent a basic health check (hematological analysis and a simple health questionnaire) and blood pressure, weight and height were recorded. Underweight and obese individuals (Body Mass Index (BMI) <18.5>30 kg/m2) were excluded. A 10 ml venous blood sample was taken for hematological measurements and anyone with abnormal values or a ferritin concentration <15 or >60 µg/L was excluded.

**Table 1:** Characteristics of volunteers.^1^

	Test meal 1 (Beads +Fe)		Test meal 2 (Fe)		Test meal 3 (Beads + Fe+ Ca)	Test meal 4 (Fe + Ca)
	Group A	Sub-group B	Group A	Sub-group B	Sub-group B	Sub-group B
Age (y)	24.2±7.9	27.5±12.4	–	–	–	–
BMI (kg/m^2^)	22.0±1.9	21.7±2.2	–	–	–	–
CRP (mg/L)	1.8±1.4	1.4±0.5	1.5±1.1	1.2±0.4	1.6±0.9	1.8±1.1
Ferritin (µg/L)	26.2±9.9	26.4±10.5	20.0±9.4	18.5±8.4	24.3±19.0	18.4±12.9
sTfR (mg/L)	6.0±3.0	6.3±3.1	6.3±2.9	6.3±2.3	7.0±2.6	6.6±2.1
BI (mg/kg)	3.9 ±2.2	3.8±2.6	2.5±2.3	2.3±2.1	2.3 ±3.5	1.5±3.6

1 Values are means ± SD.

BMI, Body Mass Index; CRP, C-reactive protein; sTfR, soluble transferrin receptor; BI, body iron.

**Figure 2:**
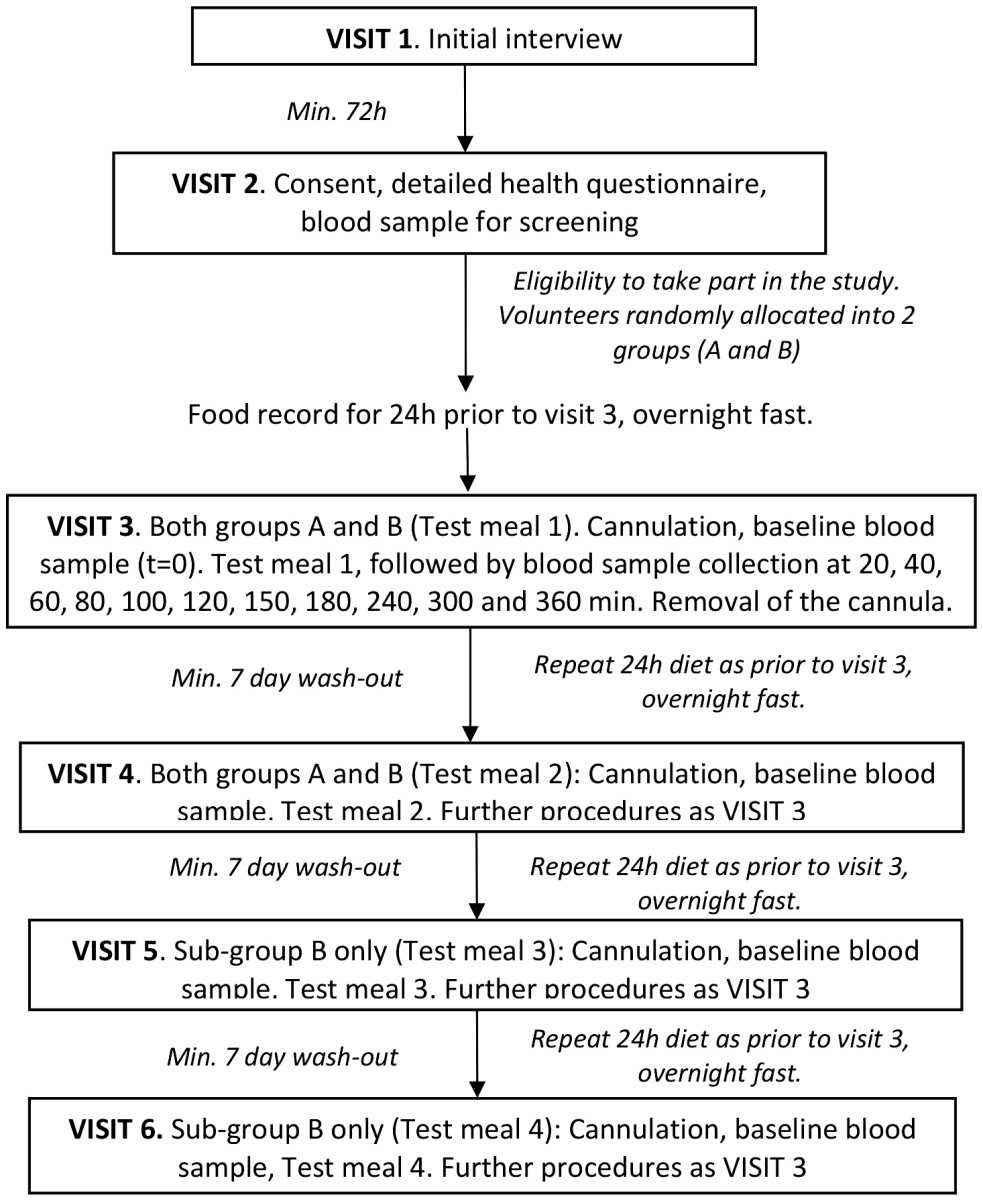
Study protocol and time-lines.

The study was a randomised, single blinded, cross-over trial (Figure 2). The protocol for this trial and supporting CONSORT checklist are available as supporting information, see Protocol S1 and Checklist S1. Test materials were given after an overnight fast and iron appearance in the serum was measured over 6 h [15], [16], [17] employing a method that has been previously validated for quantifying iron absorption [18]. Participants acted as their own controls and were randomly allocated into two groups, Group A (n = 15 volunteers) and sub-group B (n = 9 volunteers). Randomization was undertaken by a third party unconnected with the study using a randomization generator (www.randomization.com). Randomisation was generated for 16 sequential volunteers, and each random sequence generated was placed in order in a sequentially numbered (1–16), opaque, sealed envelope. As each volunteer was recruited onto the study, the appropriately numbered envelope was opened by the study scientist to reveal the assignment to group A or B. A total of 34 subjects were assessed for eligibility, 14 were excluded due to not meeting inclusion criteria, 1 volunteer declined to participate. 19 volunteers were randomized to the treatments out of which 2 were withdrawn due to the medical reasons and 1 withdrew due to the relocation. One participant was lost to follow up due to relocation. A total of 15 volunteers completed the study which started on 24^th^ April 2012 and finished on 6^th^ September 2012.

The data were subjected to compartmental modelling to calculate percentage iron absorption [18]. Group A participated in 2 out of the 4 tests, whereas sub-group B participated in all 4 tests. Each test was separated by a minimum 7 d wash-out period (Figure 2). The tests were statistically powered to ensure that the minimum number of study participants was involved in order to address the research question.

The preparation of iron-containing alginate beads took approximately 32 h, and was timed such that the beads were ready for consumption by the participants on the morning of the study day as they could only be stored for a short time in order to be safe for human consumption. Four alginates differing in mannuronic to guluronic acid ratio were previously investigated for their cross-linking capacity of ferrous gluconate in the presence of calcium chloride. Results supported the hypothesis of competitive binding between iron and calcium in alginate gel beads [19], [20]. Iron incorporation into the alginate beads was variable (1.03 mg iron/g wet bead ±SD 0.11), therefore sub-samples of every batch of beads were analysed for iron by atomic absorption spectroscopy. It was not possible to randomise the sequence of test meal administration because for every individual the dose of iron in the ferrous gluconate capsules had to be identical to the dose in the alginate beads.

The beads were prepared at the Institute of Food Research, Norwich, UK by dissolving alginate powder (Flavicans HV; 315KDalton, with D-mannuronic to L-guluronic acid ratio of 35/65 and viscosity 1% 350 cp; Danisco, DK) in distilled water. The alginate solution (0.5% (w/v (weight per volume)) was subsequently passed through an encapsulator (‘Startup’; EncapBioSystems Inc, Switzerland) where the solution was broken into equally sized beads by vibrations. The alginate droplets were then collected into a solution containing ferrous gluconate (0.1 M) and calcium chloride (0.1 M) and left to soak for 20 h at 4°C in order to crosslink and saturate with iron. Beads were then filtered for 5 min through Whatman (grade 4) filter paper and rinsed with distilled water.

Cola flavoured jelly (to mask the metallic taste of iron) was selected as a test meal, based on research conducted by Collings *et al* [21] that established that a cola drink did not affect iron absorption. Cola-flavoured jelly, into which the beads were mixed for consumption by the volunteers, was prepared by heating 200 ml of Coca-Cola in a microwave, adding 10 ml of Diet Cola flavoured drink concentrate (Sodastream, Lakeland) and 5 g of gelatine (Dr. Oetker, UK) and mixing until the gelatine was fully dissolved. Once cooled, 200 ml of the liquid jelly was poured into a disposable plastic cup and allowed to set in a refrigerator at 4°C.

Test meals were comprised of 200 g cola jelly and 200 ml of Diet Cola drink. Test meal 1 (iron in alginate beads) contained approximately 21 mg of iron as ferrous gluconate, (a quantity sufficient to perturb normal serum iron concentrations [16]), as iron-saturated alginate beads and 3 gelatine capsules (Distinctive Medical, UK) that contained approximately 50 mg dextrose each (acting as placebo). Test meal 1 (iron-containing alginate beads) also contained a calcium:iron ratio of 2.7∶1.0 as calcium was needed in the production of the beads. Test meal 2 (iron in the capsule) contained approximately 21 mg of iron (ferrous gluconate) in a gelatine capsule and 2 placebo capsules (each containing 50 mg of dextrose), and no added calcium. A sub-set of volunteers (Group B) underwent two further tests: test meal 3 (iron in alginate beads plus 600 mg calcium) with iron containing alginate beads, 2 capsules with 300 mg of calcium phosphate each and 1 placebo capsule (calcium:iron ratio 13.6∶1.0) and test meal 4 (iron in the capsule plus 600 mg calcium) with iron in the capsule and 2 capsules with 300 mg of calcium phosphate each (calcium:iron ratio 11.0∶1.0). All participants and clinical staff were blinded to the treatments.

On the day before the first test meal participants recorded all food and drinks in a food diary and consumed exactly the same diet every pre-test day. They began an overnight fast from 22:00 h and attended the UEA Clinical Trials Unit at 08.00 h. An intravenous cannula was inserted into a peripheral vein and a 5 ml baseline blood sample was taken. The test meal was consumed and 5 ml blood samples were taken at 20, 40, 60, 80, 100, 120, 150, 180, 240, 300 and 360 min. The cannula was flushed with sterile normal saline between blood samples to maintain its patency. During the 6 h cannulation period volunteers had free access to drinking water. There were no adverse events or serious adverse events reported.

All blood samples were measured for total serum iron; baseline blood samples were also analysed for serum ferritin and soluble transferrin receptor. Following blood collection into trace element- and EDTA-free tubes (BD Vacutainer, UK) the samples were allowed to stand at room temperature for a period of 30–45 min and then centrifuged at 500 g. The serum was removed and stored in microtubes (Sarstedt, Leicester, UK) at −80°C. Total serum iron was measured in duplicate using the Quantichrom (BioAssay Systems, USA) iron assay kit. Serum ferritin was analysed using a ferritin enzyme immunoassay kit (Ramco, Stafford, Texas, USA), with a mean CV of 10% (duplicate measurements). Serum soluble transferrin receptor (sTfR) was analysed using TfR enzyme immunoassay kit (Ramco, Stafford, Texas, USA), with a mean CV of 4% (duplicate measurements). In order to ensure comparability between the TfR enzyme immunoassay kit used in this study and those used in other publications the World Health Organization's (WHO) reference reagent (recombinant soluble transferrin receptor (rsTfR) National Institute for Biological Standards and Control, Potters Bar, Hertfordshire, UK) at two different concentrations, was measured on each sTfR plate with a mean value (n = 11) of 306.7 (±SD 45.5) mol/L.

C-reactive protein (CRP) concentration was measured in order to establish whether there was any low grade inflammation or infection which would affect iron metabolism. CRP measurements were performed at an accredited pathology laboratory at the Pathology Department of the Norfolk and Norwich University Hospital, Norwich, UK, and samples from any individuals with values>6 mg/L were excluded from the analysis.

As hemolysis could interfere with the Quantichrom iron assay, the optical densities of all serum samples were compared with standard serum (TCS Biosciences, Buckingham, UK) at hemoglobin peak wavelengths of 415, 417, 540, 570, and 575 nm. Hemolysis was detected in 9.5% of the samples, and these were excluded from the analysis.

Percentage iron absorption of the dose administered was calculated as follows:

Given a time of 3–6 h post-administration of the oral dose (dose_oral_) the rate of infusion R was calculated from the serum iron concentration
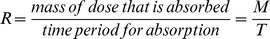
(1)


Given a volume of distribution V, and a rate constant of elimination from the compartment k, the concentration in the compartment was approximated to:
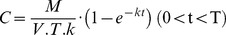
(2)

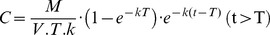
(3)


By fitting the equations to the concentration data, M, T and k were calculated.

The fractional absorption from the oral dose was then:
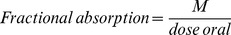
(4)


### Ethics Statement

The human study was approved by the University of East Anglia Faculty of Medicine and Health Research Ethics Committee on 31st November 2011 and was conducted according to the principles expressed in the Declaration of Helsinki. Written informed consent was obtained from every participant. The study was registered at www.clinicaltrials.gov as NCT01528644.

### Caco-2 Cell Studies

We reported previously that both alginate solutions and iron-containing beads enhance iron bioavailability *in vitro* using the Caco-2 cell model [12]. Following the human study, further *in vitro* experiments were undertaken to examine reasons for the discrepancy between the results of the human study and the previous *in vitro* results [12]. In particular, we thought it important to repeat the Caco-2 cell studies using beads prepared in exactly the same way as those used in the human study, namely washing them after preparation to remove iron adhering to the outside of the beads, mixing into cola jelly and, where appropriate, adding calcium to achieve the same calcium: iron molar ratio. It was not, however, possible to replicate the iron content of the beads exactly due to variations in iron accumulation when the beads were soaked in the iron and calcium bath.

Caco-2 cells (HTB-37) were obtained from American Type Culture Collection (Manassas, VA, USA) at passage 18 and cultured in Dulbecco's modified Eagle's media (DMEM, ATCC, USA); supplemented with 10% fetal bovine serum (ATCC, USA), 4 mM L-glutamine, 5 ml 5000 U/ml penicillin-streptomycillin solution (both from Gibco, Paisley, UK) and 5 ml non-essential amino acids 100X (Sigma, Gillingham, Dorset, UK) maintained at 37°C in an incubator with humidified atmosphere consisting of 5% carbon dioxide and 95% air. The cells were seeded onto collagen-coated 6 well plates (Greiner, Stonehouse, UK) at a density of 4.75×10^4^, and suspended in 2 ml of supplemented DMEM, which was replaced every 2 days. Cells between passages 29–34 were used for experiments at 13 d post seeding. 24 h prior to experimentation cells were switched to serum-free medium. The average Caco-2 cell protein was 0.18 mg ±SD 0.032 per cm^2^. The average ferritin concentration in cells not exposed to any iron treatment (only to digests) was 3.8 ng/mg total protein ±SD 0.7.

Alginate beads with cola jelly (and added calcium solutions in 0.1 M hydrochloric acid, where appropriate) and the positive control (ferrous gluconate), were subjected to a simulated gastrointestinal digestion prior to application onto a dialysis membrane placed above the Caco-2 cell monolayer [12]. Briefly, the samples were heated to 37°C and first underwent simulated gastric digestion with pepsin for 1 h at pH 2, and then the pH was gradually adjusted to 6.7 and pancreatin and bile extract were added to simulate digestion in the upper small intestine. The digest was applied onto the dialysis membranes placed above the Caco-2 monolayer and incubated on a rotating table for 2 h at 37°C. After 2 h the digestate was removed from the upper compartment and the Caco-2 cells incubated for further 22 h, at which point the media was aspirated from the 6 well plates and the cells were washed twice with 2 ml Phosphate-Buffered Saline. Subsequently, deionized water was applied to each well and the Caco-2 cells were scraped off using an inverted 200 µl pipette tip. The cell suspension from each well was transferred into 5 ml sample tubes and sonicated on ice, 3 times for 5 sec, using a probe sonicator. Following sonication, cell suspensions were transferred to pre-labelled 2 ml sample tubes and stored at −20°C. Sonicated cell lysates were defrosted and ferritin measured using a spectroferritin ELISA assay (Ramco, Stafford, Texas, USA). Total protein was quantified using a Pierce BCA protein assay (Fisher Scientific, Loughborough, Leicestershire, UK). Uptake of iron by Caco-2 cells was assessed from the ferritin content (ng/mg total protein).

### Statistical Methods

In order to test for an effect of alginate on iron absorption from ferrous gluconate a minimum of 12 volunteers was required to detect a difference of 2.5% at a significance level of 0.05 for 80% power. In order to test whether the interaction between alginate or ferrous gluconate and additional calcium (600 mg) affected iron absorption, a minimum of 8 volunteers was required to detect a difference in iron absorption of 3.3%, assuming a power of 80%, a level of significance of 0.05 and standard deviation of differences (within pairs) of 2.8%. Paired, two-tailed Student's t tests were used for Group A and Sub-group B to determine significant differences in iron absorption. This analysis was carried out in R version 3.0.1 [22]. For the cell studies, the statistical analysis was performed using SPSS Inc USA (version 16.0.0); 2-factor ANOVA with Tukey HSD post-hoc tests were conducted to examine pairwise differences on power-transformed data.

## Results

There were no significant differences between the subjects' characteristics in the two groups in any of the measured variables and no effect of time on serum ferritin and soluble transferrin receptor concentrations (Table 1).

**Figure 3:**
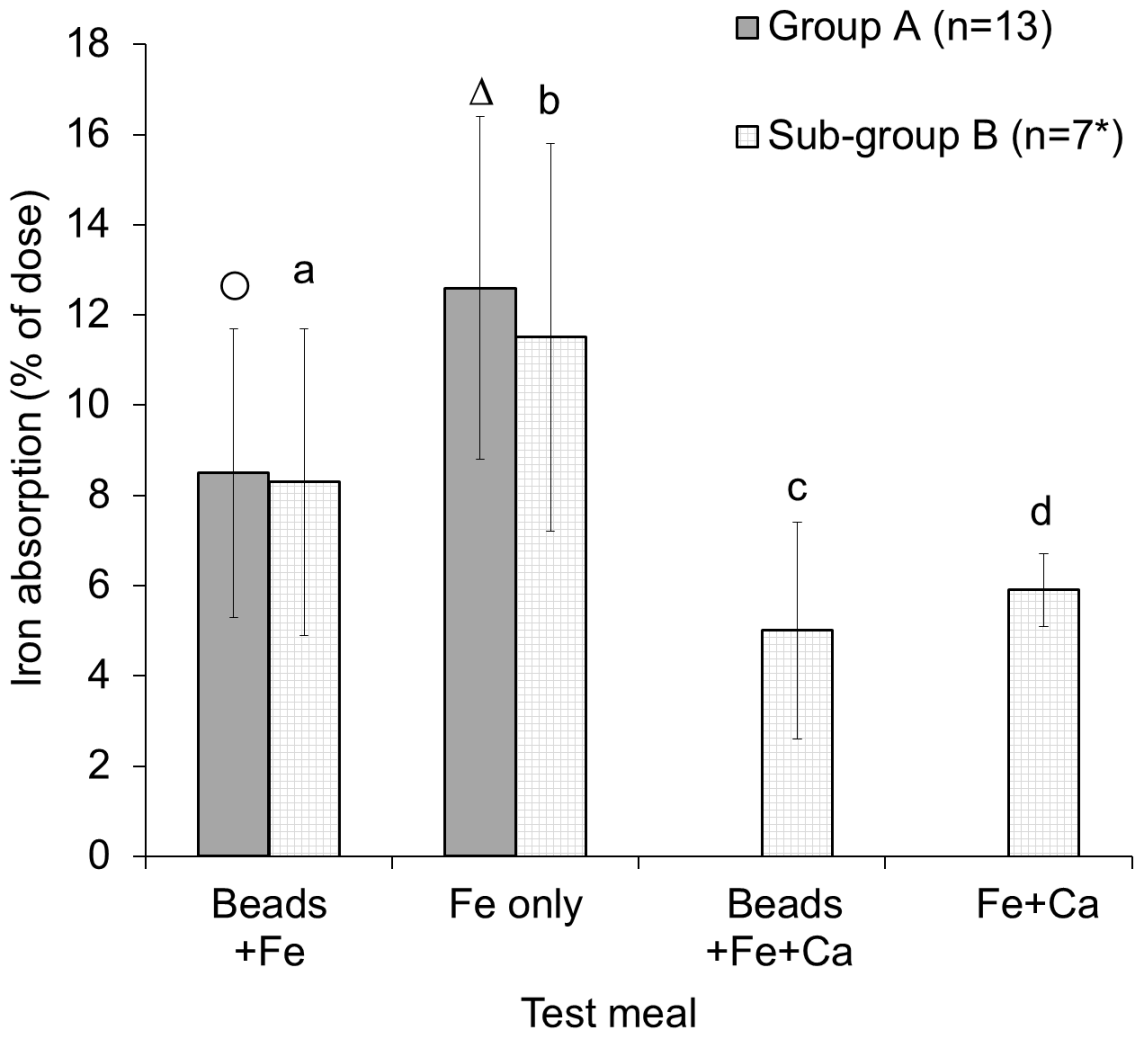
Iron absorption (% of dose). Mean values ± SD. Group A, n = 13; sub-group B, n = 7* (*with the exception of comparison between test meal 3 and 4 where n = 5). Group A means without a common symbol (○, Δ) are significantly different (*p* = 0.003). Sub-group B means without a common letter (a, b, c) are significantly different (*p*<0.05).

Iron absorption from the test meals is shown in Figure 3 (individual data in Tables S2 and S3). In several volunteers there were too many hemolysed serum samples to calculate iron absorption, so these data points are missing. There were complete sets of iron absorption data for 13 individuals in Group A (2 tests) and 6 individuals in sub-group B (all 4 tests). However, a further volunteer from sub-group B was removed because of an elevated CRP concentration. There were 7 complete sets (n = 7) of iron absorption data for Test meal 1 vs 3 and for Test meal 2 vs 4. Alginate significantly reduced mean iron absorption from 12.6% when given as ferrous gluconate (Test meal 2) to 8.5% when given as alginate beads (Test meal 1; p = 0.003, n = 13). As predicted, 600 mg calcium had a strongly inhibitory effect on iron bioavailability; ferrous gluconate absorption fell from 11.5% (Test meal 2) to 5.6% (Test meal 4; p = 0.014; n = 7). Calcium also reduced iron absorption from the alginate beads, from 8.3% without calcium (Test meal 1) to 5.2% with calcium (Test meal 3; p = 0.009; n = 7).

In the previous Caco-2 cell studies alginates enhanced iron uptake into the cells. However when alginate beads were produced containing a lower iron concentration (with an inevitable increase in calcium) it resulted in a lower ferritin response [12]. Additional Caco-2 cell studies were therefore undertaken to try to find an explanation for the difference between the *in vitro* and *in vivo* findings.

**Figure 4:**
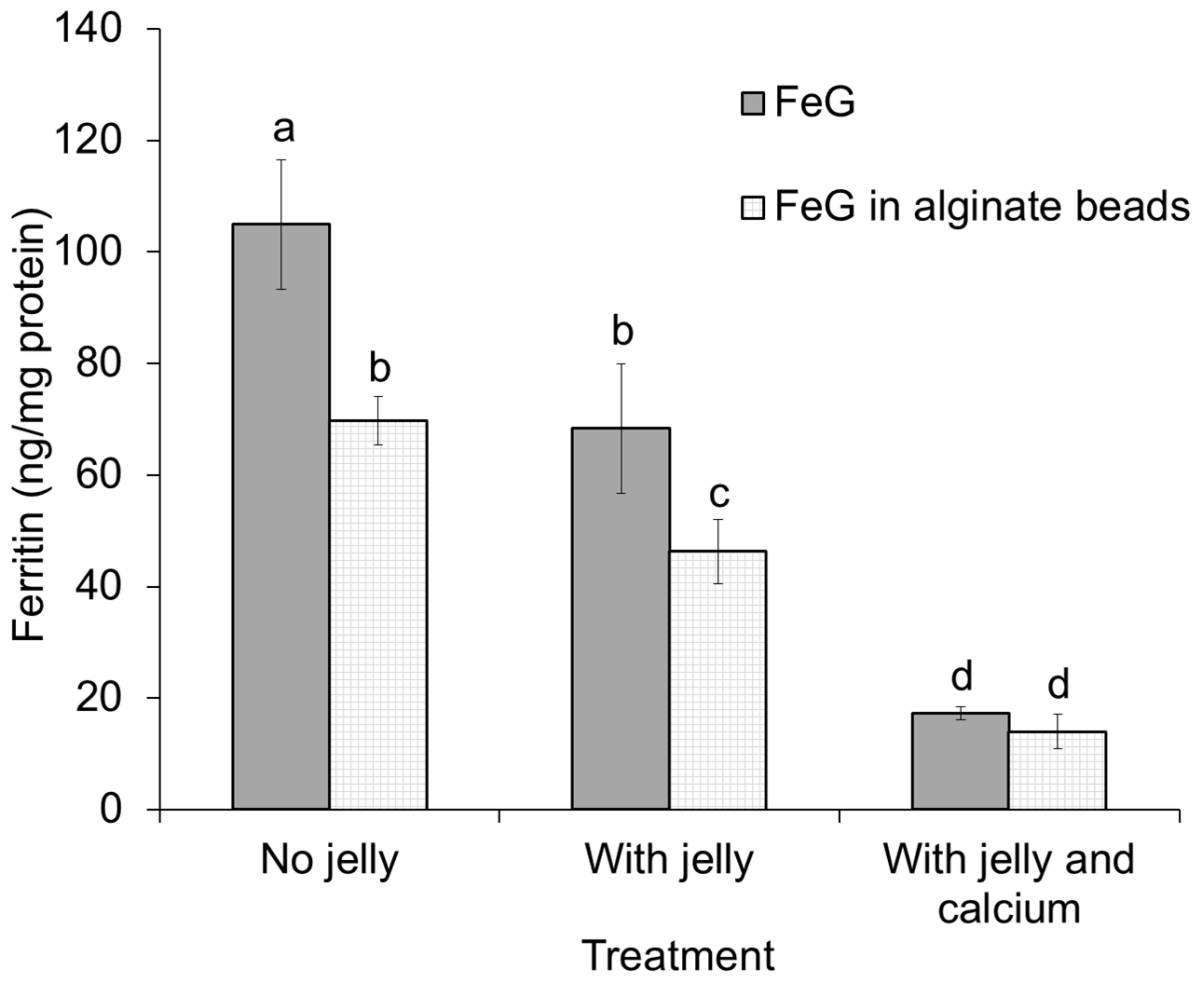
Iron uptake in Caco-2 cells exposed to ferrous gluconate (FeG) alone or in alginate beads, with or without cola jelly test meal, or with cola jelly test meal in the presence of calcium phosphate. Bars represent mean ±SD ferritin concentration (ng/mg total protein) after treatment with 102.3 µmol/L FeG (n = 6 for all treatments). Bars without a common letter (a, b, c, d) are significantly different, p<0.005 (a>b>c>d). Ferritin concentration in cells with no treatment was 7.6±SD 1.0.

An attempt was made to replicate the beads that were used in the human study. When Caco-2 cells were exposed to digestate from a simulated gastrointestinal digestion of alginate beads containing ferrous gluconate, with and without cola jelly, both alginate and cola jelly significantly reduced iron uptake into the cells (by 34%, *p* = 0.009 and 35%, *p* = 0.003 respectively). The combination of cola jelly and calcium produced a very low ferritin response, 16.5% (*p*<0.001) of that observed with ferrous gluconate alone (Fig. 4). The results show that the alginate bead test meal, similar to that used in the human study, reduced iron availability. Practical constraints made it impossible to replicate the composition of the beads exactly; the calcium and iron content of the beads used for the human study were 1.96 and 1.04 mg/g whereas for the cell study they were 1.62 and 1.72 mg/g respectively.

## Discussion

The human study results were not consistent with the earlier *in vitro* results which showed that alginate enhanced iron uptake in epithelial cells [12]. Calcium exerted a very powerful negative effect on iron absorption, which was greater with ferrous gluconate solution (51% decrease) than with the alginate beads (37% decrease) due, presumably, to the fact that alginate had already reduced iron absorption by 33%. The possible reasons for the conflicting findings between the earlier cell studies and the human study might be the higher level of calcium in the beads plus cola jelly test meal, and also the lower iron content of the beads following washing to remove iron adhering to the outside of the beads (the rinsing step was undertaken to make the preparation more palatable for the volunteers).

Calcium has a strongly inhibitory effect on iron absorption *in vivo* [23] and *in vitro* [24]. One proposed explanation for the enhancing effect of alginate in the previous Caco-2 cell studies was that it bound calcium in the culture medium, thereby reducing the inhibitory effect of calcium on iron uptake. In the human study, the calcium dose selected was 600 mg, because this quantity of calcium was required to achieve a calcium:iron molar ratio of 11∶1 with the minimum dose of iron required to produce a measurable serum response (21 mg). A dose-dependent inhibitory effect of calcium on absorption of iron has been reported for molar ratios ranging from 11∶1 (40 mg calcium, 5 mg iron) to 83∶1 (300 mg calcium, 5 mg iron) [25], although more recently a higher threshold molar ratio of 220∶1 (800 mg calcium, 5 mg iron) was reported [26]. Our study demonstrates an inhibitory effect of calcium at a molar ratio of 11∶1 (Figure 3, iron alone 11.5%, iron plus calcium 5.6%, p = 0.014, n = 7).

There is one other human study in which the effect of alginate on iron absorption was measured [13]. Six ileostomy patients were given a low-fiber diet (providing 1,756 mg calcium/d and 7 mg iron/d) with or without a daily supplement of 7.5 g sodium alginate. In 5 of the 6 subjects alginate resulted in a lower apparent absorption of iron, although the results were not statistically significant due to high inter-individual variation and insufficient power. However, as the diets were similar apart from the addition of sodium alginate, the results suggest an inhibitory effect of alginate.

Bosscher *et al.* [27] reported that the availability of iron and zinc from infant formulas increased with the addition of alginic acid when investigated using an *in vitro* dialysis model with a preliminary intraluminal digestive phase. The nature of the binding of alginate to iron is unknown. Results from circular dichroism and zeta potential experiments indicate that the interaction at pH 3.5 (relevant to gastric conditions) is through a site-binding model, with an estimated 66 sites per molecule of sodium alginate [10].

## Conclusions

The results of the human study and the Caco-2 cell experiments demonstrated that alginate beads are not a suitable delivery vehicle for food iron fortification in diets consumed in industrialized countries. The inhibitory effect of alginate beads appears to be linked to calcium; but as calcium is needed for a stable alginate gel to be formed low-calcium beads are not a viable alternative. However, with high phytate diets, it is possible that alginate might bind iron sufficiently tightly to protect it from binding to phytate (which prevents it being absorbed), and might therefore deliver a useful quantity of absorbable iron. This is the proposed action of iron-EDTA and it would be worth investigating whether alginate can perform a similar function.

## Supporting Information

Protocol S1 **Trial protocol.**
(PDF)

Checklist S1 **CONSORT checklist.**
(DOCX)

Table S1 **Individual data for BMI, gender, C-reactive protein, serum ferritin, soluble transferrin receptor and body iron.**
(XLSX)

Table S2 **Individual data for ferrous gluconate absorption with and without alginate.**
(DOCX)

Table S3 **Individual data for ferrous gluconate absorption with and without alginate and calcium.**
(DOCX)

Form S1 **Consent form.**
(DOC)

Form S2 **Health questionnaire.**
(DOC)

Form S3 **Adverse events form.**
(DOC)

Form S4 **Subject record sheet.**
(DOCX)

Form S5 **Research Ethics application.**
(PDF)

Form S6 **Ethics approval letter.**
(PDF)
