# Supplementary material for: Alginate Inhibits Iron Absorption from Ferrous Gluconate in a Randomized Controlled Trial and Reduces Iron Uptake into Caco-2 Cells
Source: PLoS One. 2014 Nov 12;9(11):e112144. doi: 10.1371/journal.pone.0112144 (PMC4229116; doi:10.1371/journal.pone.0112144)
Supplement: Protocol S1 — Trial protocol. (PDF) [file pone.0112144.s001.pdf]

Study to measure the absorption of iron from ferrous gluconate incorporated into alginate beads

Short title: Iron & Alginate Study

Protocol

Academic Supervisor: Prof. Susan Fairweather-Tait

Chief Investigator: Anna Wawer

BBSRC grant reference number: BB/G0055833/1

Version 1, 23/9/2011

Contents

1. Summary 2
1.1. Aims 2
1.2. Approach 2
1.2.1. Study design 2
1.2.2. Study Population 8
2. Scientific Background 8
3. Objectives 11
4. Hypothesis 11
5. Recruitment Strategy 11
6. Volunteer Screening 12
6.1. Visit 1. Initial Interview 12
6.2. Visit 2. Consent and screening 13
7. Inclusion / Exclusion Criteria 15
8. Study Procedures 17
8.1. Visit 3: First experimental day 18
8.2. Visit 4: second experimental day 20
8.3. Visit 5: third experimental day 21
8.4. Visit 6: fourth experimental day 21
9. Iron Dose Preparation 22
9.1. Ferrous gluconate preparation 22
9.2. Preparation of iron containing alginate beads 22
10. Test meal preparation and consumption 23
11. Analytical methods 23
11.1. Serum sample preparation 23
11.2. Serum sample analysis 24
12. Confidentiality 24
12.1. Storage of the data 24
13. Volunteers expenses / payments 25
14. Statistical analysis 26
14.1. Statistical power 26
14.2. Statistical method 26
14.3. Data analysis 27
15. References: 29

1. Summary

1.1. Aims

The aim of this study is to assess the effect of alginate on iron absorption. This will be achieved by measuring the absorption of iron, in the form of ferrous gluconate encapsulated in alginate beads compared with non-encapsulated iron i.e. without alginate. In addition, the modulating effect of calcium (a known inhibitor of iron absorption) will be measured since calcium also forms strong complexes with alginate. The development and production of the iron beads is novel research currently being undertaken by colleagues at the Institute of Food Research. Therefore, this study will employ standard analytical techniques to evaluate iron absorption in the context of a novel iron delivery system.

1.2. Approach

When combined with ascorbic acid, ferrous gluconate is an iron compound that has been reported to be an effective food fortificant, which may reduce the prevalence of anaemia[1] In order to maximise iron absorption *in vivo*, it would be advantageous to employ a delivery system that will protect the iron from adverse interactions in the gut lumen and deliver iron directly to the duodenum. This study aims to investigate the effectiveness of using an environmentally-responsive impermeable alginate barrier to deliver ferrous gluconate to the duodenum, thus maximising its availability for absorption.

1.2.1. Study design

The study design and outline are presented in Figures 1 and 2. This study is designed as a randomised, single blinded, cross-over trial with participants randomly allocated into two groups: A (n= 8 volunteers) and B (n=8 volunteers). For statistical power of this study please refer to section 14.1. By administration of test meals 1 and 4 scientist will investigate the possible protective effect of alginate on iron absorption (primary outcome). By administration of test meal 2 and 3 the research team will investigate whether alginates can bind calcium (a well-known iron inhibitor) *in vivo,* thus diminish the potential negative effect of calcium on iron absorption (secondary aim).

Only half (8) of the volunteers will undergo treatments including calcium. Therefore group A will undergo all 4 tests, whereas group B will undergo 2 out of 4 tests.

This study will be undertaken to compare the absorption of iron (approximately 20mg) added to cola jelly (200ml) as ferrous gluconate incorporated into alginate beads (approx. 22.2g) (test meal 1), with iron at the same dose given as ferrous gluconate in a commercially available capsule along with a diet cola drink (200ml), (test meal 4). The third test for group A only (test meal 3) will involve consuming iron incorporated into the alginate beads (approx 22.2g) added to cola jelly (200ml), followed by 2 capsules, each filed with 300mg commercially available calcium phosphate powder (Freeda vitamins, USA, http://www.freedavitamins.com/index.php?p=product&id=120&parent=10) (600mg in total) in the form of calcium phosphate. Cook *et* al[3] has shown that this dose and form of calcium was the most effective in iron inhibition. The addition of calcium will determine whether the alginate barrier can reduce or prevent the inhibitory effect of calcium on iron absorption. The fourth test (test meal 3) will involve consuming cola jelly (200ml), a ferrous gluconate capsule (approx. 20mg) and two calcium phosphate capsules (300mg of calcium in each capsule) followed by a diet cola drink (200ml).

The development of the iron-containing beads is novel on-going research and consequently the preparation of the iron-containing alginate beads imposes certain limitations on the proposed study design. Currently, iron incorporation into the alginate beads is within 15% of that predicted. Analysis of the iron (atomic absorption spectroscopy) incorporated into the alginate beads is a three day procedure, and long term storage method for the beads has not yet been established. These limitations mean that the sequence of the test meals cannot be randomised (Fig.1). Iron containing alginate beads will be prepared for each volunteer approx. 48 hours before the test meal and stored in a refrigerator in a food grade kitchen at 4ºC. Sub-samples of each batch of beads administered to each volunteer will be collected and their iron content analysed using atomic absorption spectroscopy. Once the exact content of iron present in the beads administered to each specific volunteer in Test meal 1 and 2 is known (and recorded), exactly the same amount of ferrous

gluconate will be weighed into a capsule and administered to that volunteer in a subsequent test meal (Test meal 4 and 3 respectively) after a minimum 7 day wash-out period.

For each test meal an oral dose of iron (approximately 20mg), sufficient to perturb the normal serum iron concentration[2], will be given to apparently healthy adult volunteers after a 10 hour (overnight) fast.

All test meals will be prepared by a trained (holding a level 2 award in food safety in catering) member of the scientific staff in the Clinical Research Trial Unit kitchen. The four test meals will be as follows:

- Test meal 1 for all volunteers (group A and B): 200ml cola jelly prepared from 200ml cola (Coca-Cola), 10ml of commercially available Diet Cola flavoured Drink Concentrate (Sodastream, Lakeland) and gelatine (Dr. Oetker) gelatine (from animal source, Dr. Oetker, UK) with the addition of ferrous gluconate (approx 20mg +/- 2mg) in alginate beads (approx. 22.2g) followed by 200ml of diet cola drink (Coca Cola) with 3 placebo capsules (filled with 50mg commercially available dextrose powder, ‘Glucose Dextrose Powder’, PharmacyNearU.com) acting as placebos for calcium and an iron capsules.)
- Test meal 2 for 8 volunteers only (group A): 200ml cola jelly prepared from 200ml cola (Coca-Cola), 10ml of commercially available Diet Cola flavoured Drink Concentrate (Sodastream, Lakeland) and gelatine (from animal source, Dr. Oetker) with the addition of ferrous gluconate in alginate beads followed by 200ml of a diet cola drink (Coca Cola) with 2 commercially available capsules containing calcium phosphate, (Freeda vitamins, USA) (300mg of calcium in each capsule, in total 600mg of calcium)[3] and one placebo capsule in place of the iron capsule.
- Test meal 3 for 8 volunteers only (group A) : 200ml cola jelly prepared as above without iron containing alginate beads(approx. 22.2g), 2 calcium capsules (as above) and 1 ferrous gluconate capsule (prepared exclusively for each volunteer to match the iron content in the alginate beads administered in test meal 2) followed by 200ml diet cola drink.

- Test meal 4 for all volunteers (group A and B): 200ml cola jelly prepared as above, 1 ferrous gluconate capsule (prepared exclusively for each volunteer to match the iron content in the alginate beads administered in test meal 1) followed by two dextrose (50mg of dextrose in each capsule) placebo capsules in place of the calcium capsules and 200ml of diet cola drink.

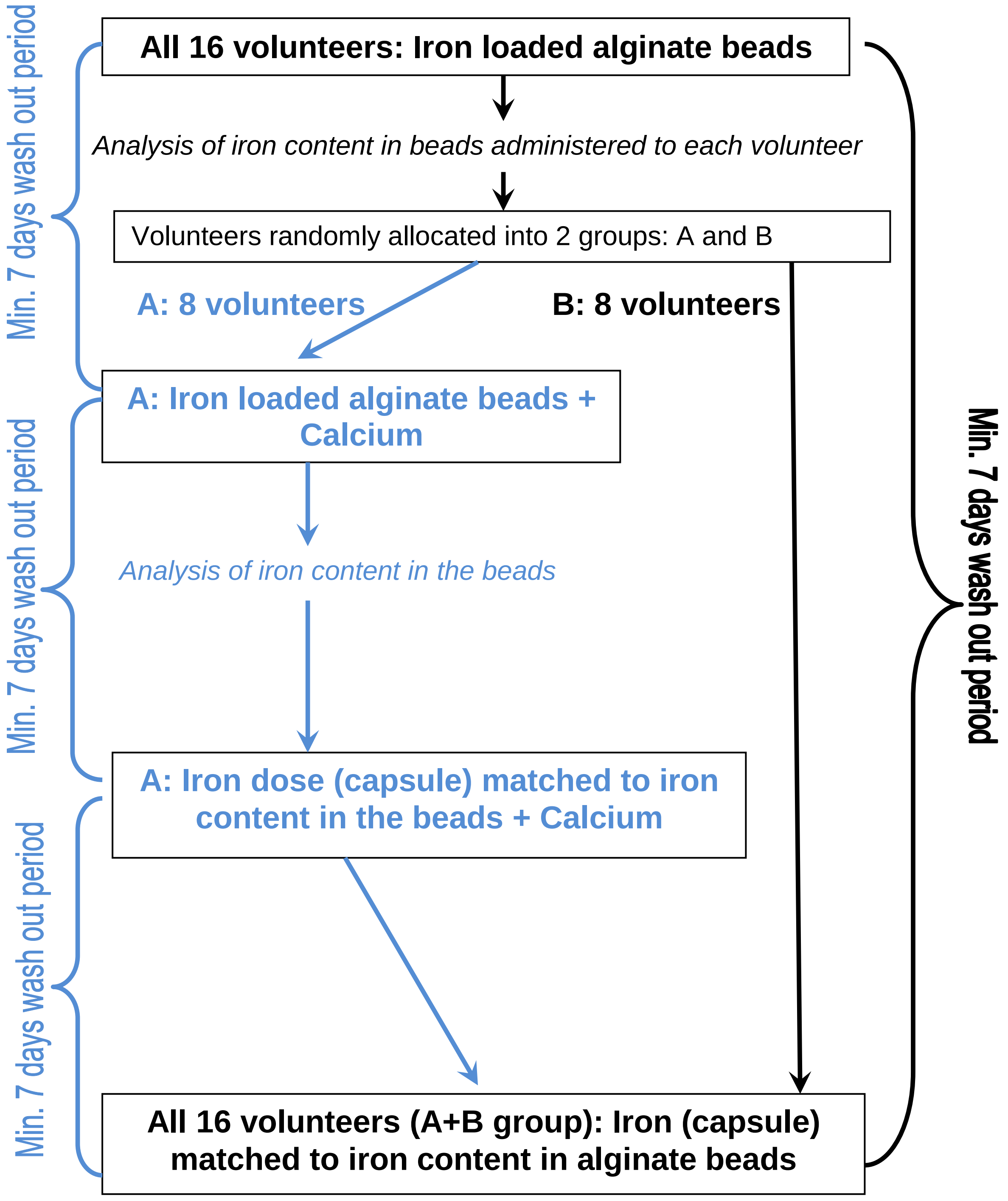

Figure 1. Study design

All appointments which volunteers will attend to (starting from initial interview through the screening and test visits) will take place in Clinical Research and Trials Unit (CRTU) based in the Norwich Medical School at the University of East Anglia. The CRTU provides all the required facilities and resources for performing human trials.

A trained nurse based in the CRTU will undertake the following clinical procedures. On each test meal day an intravenous (i.v.) cannula will be inserted into an appropriate peripheral vein in one of the volunteer's arms. The cannula will remain in situ for six hours. A baseline blood sample will be taken prior to consuming the test meals. Further blood samples (5ml each time) will then be taken 20, 40, 60, 80, 100, 120, 150, 180, 240, 300 and 360 min after ingestion of the test meals (described above) and collected into appropriate tubes for the preparation of serum. Serum iron concentrations will be measured in each sample using a colorimetric assay. Figure 2 provides a flow chart of the study outline.

Absorption will be calculated from the area under the iron serum appearance curve using a compartmental modelling approach[4, 5]

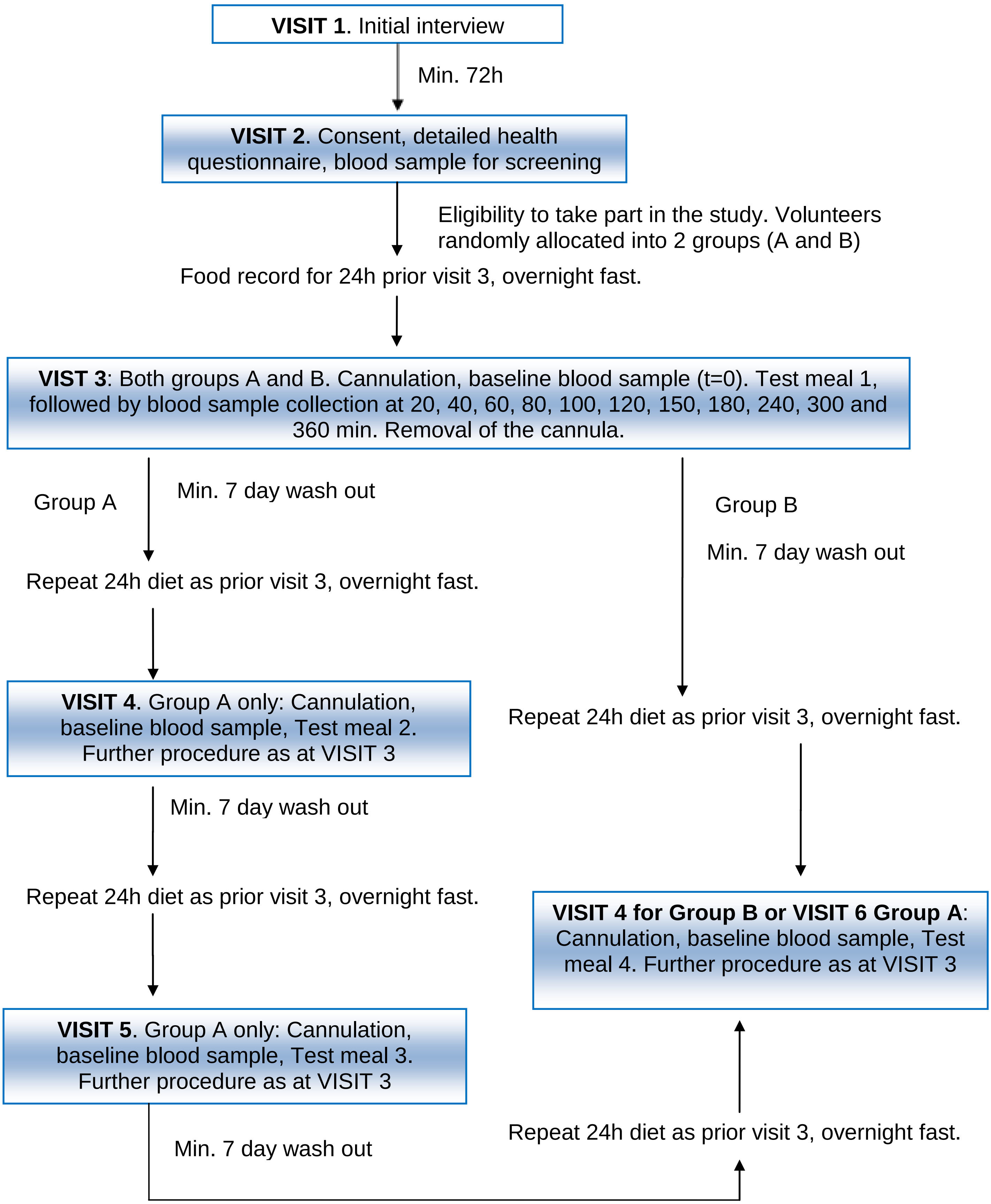

Figure 2. Flow chart of study outline.

1.2.2. Study Population

Volunteers will be recruited from a target population of women of child-bearing age (18-45y) and men who are regular blood donors (18-65y) as these individuals generally have iron stores at the lower end of the normal range. Iron absorption is more efficient when ferritin stores are below <60µg/L[6] and therefore this will maximize the sensitivity of the absorption test.

Female volunteers who decide to take part in the study will be asked by a study scientist at the start of each experimental day if they are menstruating and if they are not, then they will be asked how many days ago menstruation has completed. This information may help to explain potential fluctuations in ferritin levels and potential differences in iron appearance in the serum during different experimental days. Menstrual iron losses may affect biomarkers of iron status such as ferritin[7] which may in turn affect iron absorption.

2. Scientific Background

According to the World Health Organisation (WHO) more than 2 billion people[8] worldwide suffer from anaemia. Iron deficiency anaemia accounts for 50% of all anaemias[9] with infants, children and pre-menopausal women at most risk.

Both in developing and developed[10] countries the consequences of anaemia result in significant health problems and economic cost.

Nutritional iron deficiency is the consequence of dietary iron being insufficient to meet physiological needs[8]. Depending on the presence of dietary enhancers and inhibitors, non-haem (inorganic) iron absorption from the diet ranges between 1% and 15%[11]. Therefore the cause of nutritional deficiency is often related to low bioavailability rather than inadequate intake of iron.

There is a well-justified use of iron fortification as a strategy to prevent iron deficiency anaemia. Food fortification with iron is recognised as a sustainable and realistic way to reduce the occurrence of iron deficiency[12]. Nonetheless it is challenging to the food industry, mainly due to organoleptic changes during storage or preparation[13]. Water soluble forms of iron are considered to be

more bioavailable than non-soluble iron compounds. However, the former often cause sensory problems when added to foods, while the latter cause fewer problems but are not generally well absorbed and are therefore ineffective as fortificants. A potential strategy for overcoming this problem is the use of water soluble iron compounds protected by a water resistant barrier which will prevent potential organoleptic changes of fortified foods. Preliminary, *in vitro* work using Caco-2 cells has been undertaken to establish the most promising protective barrier. Caco-2 cells are human gastrointestinal tumour cells originally isolated from a colon adenocarcinoma in 1974[14]. The Caco-2 cell line has been shown to have long viability under culture conditions (thus reproducibility of experiments) and to share many characteristics of normal small intestine absorptive cells[15]. Due to these properties Caco-2 cells have been used in various nutritional studies as a model for the human small intestine[16]. The main advantage of using Caco-2 cells to investigate transport of micronutrients and their bioavailability is that they are derived from a human source unlike other techniques using rodent models[17]. The Caco-2 cell model can be used along with a simulated digestion phase. Before application of the food matrix onto the dialysis membrane[18] which is placed above Caco-2 cell monolayer, samples undergo simulated digestion phase which imitates the enzymatic phase of human digestion in the stomach and duodenum and associated changes in the pH[19].

Caco-2 cells combined with simulated digestion is a useful model system that attempts to imitate small intestinal conditions and is a valuable tool for screening different iron sources in order to rank them for potential bioavailability and for investigating mechanisms of nutrient absorption in humans[20,21].

In studies undertaken by the research team, addition of 1% (w/v) alginate solution was shown to have an enhancing effect on iron uptake in the *in vitro* Caco-2 cell model as measured by ferritin formation, a surrogate index of iron availability. Across 2 experiments with the use of 4 different alginates ferritin formation was on average 2.27 (±SD=0.42) fold greater in cells treated with 1% alginate solution (w/v) in the presence of iron when compared with cells treated with iron only. (For more details see Annex 1, chart A and B). Moreover, the data demonstrated that alginate beads containing ferrous

gluconate provided protection to the iron throughout the *in vitro* digestion process and delivered iron in an available form for uptake by Caco-2 cells. The protective/enhancing effect of alginate was equivalent to the enhancing effect of ascorbic acid at 1:2 (Fe:AA) ratio (For more details see Annex 1, chart C).

This possible enhancing effect of iron containing alginates occurring *in vitro* may be related to the preference of alginate to bind the calcium that is present in the cells treatment medium, thus avoiding the inhibitory effect of calcium. This hypothesis will be tested *in vivo* by the simultaneous administration of alginate beads containing ferrous gluconate and commercially available capsule with calcium phosphate powder[3].

Physico-chemical studies performed at the Institute of Food Research (IFR), Norwich have resulted in the selection of an appropriate alginate polymer to be tested *in vivo* in this study.

Alginates are natural biopolymers extracted from brown seaweed consisting of linear copolymers of (1→4) linked β-D-mannuronic acid (M) and its C-5 epimer α-L-glucuronic acid (G). With the added variable of polymer length, this class of polysaccharides exhibits a broad spectrum of physicochemical properties[22]. This variation ultimately underlies why alginates are commonplace in multiple industrial processes and are of valuable use both in medicines and the food industry. Within the food industry alginates are widely used as gelling, thickening, emulsifying and stabilising agents and thus extensively found in products such as ice creams, jams, sauces and desserts[23]. In addition alginates are also found in medicines, most notably anti-reflux medicines such as Gaviscon Advance™.

Alginates have been shown to bind divalent and trivalent cations[24, 25] and therefore may be a useful vehicle for soluble iron compounds used to fortify foods. In addition, it has also been demonstrated that alginates can form a stable complex with ferric iron over a range of different pH values[25]. On the other hand, studies carried out in ilestomy subjects[26] have shown that alginates can decrease iron absorption. However, this finding was not statistically significant probably due to a small sample size and/or the administration of alginate as a powder. In the present study alginate will be

dissolved in water at 0.5% (w/v) concentration and saturated in a calcium and iron bath during the formation of the beads.

3. Objectives

The aim of this project is to measure the effect of alginate on the absorption of iron from ferrous gluconate and any modulating effect of calcium.

Primary outcome:

- The determination of iron absorption (by measuring serum iron appearance) from iron gluconate incorporated into alginate beads compared with that from unprotected ferrous gluconate.

Secondary outcome:

- A comparison of iron absorption from ferrous gluconate incorporated into alginate beads given with calcium (iron inhibitor) and from unprotected ferrous gluconate given with calcium.

4. Hypothesis

The primary hypothesis is that the alginate in iron containing alginate beads will act as a protective barrier throughout the gastric phase of digestion, and thus will deliver more available iron for absorption into the mucosal cells of the duodenum than ferrous gluconate administered in a capsule.

In addition, the secondary hypothesis is that the alginate will bind calcium thus decrease its inhibitory effect on iron absorption.

5. Recruitment Strategy

Apparently healthy women of child-bearing age (18-45y) or male (blood donors only) volunteers (age 18-65y) with low iron stores (serum ferritin ≥15 ≤ 60µg/L) will be screened until 16 participants have been recruited. Based on previous experience with similar studies, dropouts and unsuccessful blood collection will mean that only 13 complete sets of data will be obtained for analysis. This is, however, sufficient for this study (see power calculation, section 14.1).

Participants will be recruited from the Norwich Research Park (NRP) and the surrounding Norwich area. Advertisements will be placed on the University of East Anglia (UEA) website, NRP newsletters, distributed via email to UEA staff and students (Annex 2), and placed on notice boards at IFR, the John Innes Centre (JIC), TGAC, UEA schools, local supermarkets, and other suitable locations (e.g. gyms) inviting anyone interested in receiving information about the study to contact named researchers by telephone or e-mail.

Volunteers who meet the basic inclusion criteria (section 7), will be sent a participant information sheet (PIS) (Annex 3) which will include a response form and a freepost envelope with which to reply if they are interested in participating in the study.

There are no arrangements for participants who might not understand verbal or written information given in English as the cost of translating study information would be prohibitive.

This is a small study and it is expected that sufficient volunteers fluent in English will be recruited from across the Norwich Research Park. However, should there be any doubt regarding a volunteer's understanding of what the study involves then they will not be recruited. This assessment will be made by the study scientist at the stage of discussion about the study.

6. Volunteer Screening

6.1. Visit 1. Initial Interview

Apparently healthy women or men (blood donors only) potential volunteers who respond positively to the advertisements and the PIS (via email, telephone or post) will be asked to visit the CRTU for an informal meeting to receive further information about the study. Responders will be invited to the meeting on an individual basis to ensure privacy and a study scientist will explain the details of study participation. During the meeting the study scientist will follow the interview script (annex 4), which will act as a prompt to

the scientist to ensure that the volunteer understands his/her role in taking part in this study. The script will not be followed word-for-word but will simply act as a reminder to the study scientist to make sure all aspects of the study have been fully explained to the potential participant. Volunteers who meet the basic inclusion and exclusion criteria (section 7) will be encouraged to ask any questions they may have about the study prior to making any commitment and will be given a minimum of 72 hours (3 days) to consider whether they wish to participate in the study. In addition they will be asked to select a meal (from the Catering Direct packed lunch menu) which they would like to consume after the screening visit. During the consideration period the volunteers will not be contacted by any member of the research team. If, following the period of consideration, the volunteer wishes to participate in this study they will be advised to contact the named researchers to arrange for a clinical screening visit at the CRTU. If the volunteers are very keen to participate in the study and ask to book a screening date at the meeting, this can be arranged with the appointment booked for after the minimum of 72 hours consideration period.

6.2. Visit 2. Consent and screening

Prior to being accepted onto the study, all volunteers who are willing to participate following the initial informal meeting will be clinically screened to ensure that biochemical indices fall within the standard reference ranges for age and sex. Volunteers will be invited to attend a clinical screening visit at the CRTU after an overnight fast of at least 8 hours. They will be advised to drink water during the fasting period. Potential participants will also be reminded to bring details of any regular medication or dietary supplements they take (i.e. dose taken, name of medication).

On arrival, the study scientist will go through the consent form with the volunteer and encourage any questions they may have at this stage. Volunteers will then be asked to sign a consent form agreeing to participate in the study (Annex 5). A copy of this form will be given to the volunteer to keep. Subsequently, a researcher along with an experienced CRTU nurse will

complete a basic health questionnaire (Annex 6) and collect information about weight and height. The CRTU nurse will obtain and record blood pressure measurements, pulse and BMI. A single venous blood sample (10ml) will also be taken by a CRTU nurse for assessment of iron status and to confirm lack of chronic inflammation/infection (full blood count: Wbc, Rbc, Hb, Hct, MCV, MCH, platelets, Neutrophils, Lymphocytes, Monocytes, Eosinophils, Basophils, fasting glucose and ferritin). In addition C reactive protein (CRP) assessment from baseline blood samples will be performed on each experimental day to establish if serum iron appearance could be affected by inflammation. The blood will be analysed at an accredited pathology laboratory at the NNUH, Norwich. Once all clinical procedures are completed, the volunteers will be offered and encouraged to have a meal which they selected during the screening visit.

During that meeting volunteers will also be asked to select a meal from the Catering Direct (UEA) 'Scholars packed lunch' list which they would like receive at the completion of experimental days. This information will be recorded by the study scientist.

Volunteers will be excluded if their BMI is <18.5 and >30 or their blood pressure measurements are >160/100. Volunteers will also be excluded from further participation if the serum ferritin is less than 15ug/L (indicates iron deficiency) or greater than 60µg/L (The full list of inclusion / exclusion criteria are given in section 7)

Blood screening results will be examined by an experienced CRTU nurse who will advise the study scientist if all screening parameters are within the normal range for sex and age, and therefore whether the participant can take part in the study. In addition, screening results will be forwarded to the volunteers G.P. If any of the parameters (apart from ferritin values of ≥15 ≤ 60µg/L, study specific requirement) fall outside the normal range the results will be forwarded to the study medical advisor Dr Phyo Myint, UEA. Dr Myint will then be contacted by a study scientist and asked whether the results could affect the study data or have implications for the health of the volunteer, and therefore whether inclusion, exclusion or re-screen is appropriate.

The GPs of those successfully recruited onto the study will be informed of their patient's participation in the study by letter (Annex 7) and will be sent copies of all clinical screening results (Annex 8, Annex 9). If the CRTU nurse advises the study scientist that blood screening results are abnormal, the volunteer will be asked to contact their G.P. Volunteers will be informed of flagged blood results by a telephone call and letter (Annex 11) advising them to speak to their GP to discuss the results. Non-clinical study scientists will not discuss clinical results with individual volunteers. All abnormal clinical results obtained during the study from fresh samples (not stored), that have been analysed at an accredited laboratory and which fall outside the standard reference ranges, will also be sent to the GP (Annex 10).

The volunteer will agree to this information being sent to the GP by signing the consent form.

During the screening interview the study scientist will also provide instructions on keeping a 1-day food diary to record the "pre-test diet" in preparation for the first experimental day (VISIT 3). Volunteers will be asked to repeat the same pre-test diet prior to each experimental day. In cases where any food or drink was consumed in addition to the pre-test diet or was forgotten to be eaten, volunteers will be asked to note it down in the Record of Differences Diary at the end of the food intake diary (Annex 12). During that meeting participants will also receive instructions with regard to undertaking 10 hour overnight fast. They will be encouraged to have a meal no later than 9pm on the night prior to each experimental day and aim to arrive at the CRTU at around 8:00 am the next morning.

7. Inclusion / Exclusion Criteria

The Inclusion Criteria for this study are:

- Apparently healthy women (aged 18-45) or male blood donors (aged 18-65)
- Serum ferritin value of ≥15 ≤ 60μg/L

- BMI >18.5 or < 30
- Willingness to consume jelly prepared with the use of gelatine from an animal source

The exclusion criteria for this study are:

- Diagnosed with a long-term illness requiring active treatment, e.g. diabetes, cancer, cardiovascular disease
- Currently smoking or stopped smoking in the last 12 months
- Previous or current GI disease
- Any significant co-morbidity
- Previous GI surgery
- Blood donation within the previous 3 months assuming that volume taken is above 500ml in a 4-month period
- Blood transfusion within the last 3 months
- The results of the screening tests indicate unsuitability to take part in the study
- Related to someone in the study team i.e. spouse, partner or immediate family member
- Regular prescribed medication that may interfere with iron metabolism
- Regular use of antacids and laxatives (at least once a week)
- Women who are pregnant or less than 12 months since giving birth
- Women who are breast feeding
- Vitamin supplements with or without minerals if taken more than once a week, and unwillingness to discontinue occasional use for the duration of study
- Unwillingness to discontinue use of herbal supplements for the duration of study
- Unwillingness to consume jelly prepared with animal source gelatine
- Use of antibiotics within four weeks prior to study start

- Parallel participation in another study which involves dietary interventions or sampling of blood that may increase the volume taken above 500ml in a 4-month period
- Asthma requiring treatment within the last two years
- Results of clinical screening which indicate a health problem which could compromise the well-being of the volunteer if they participated or which would affect the study data.

8. Study Procedures

Randomization

Volunteers will be assigned randomly to group A or B and they will be asked to complete up to 4 study days (4 in group A and 2 in group B). Volunteers will be unaware that if they were randomly assigned to group A it will result in completing 4 study days and that if they were assigned into group B it will result in completing 2 study days. Maintaining volunteers' unawareness will improve flexibility of pairing them for experimental days. After completing 2 study days, volunteers from group B will be contacted, thanked for their involvement and informed that their participation in the study is complete as all the required data have been collected. The PIS will contain details advising potential study participants that they will be randomly selected to take part in either two or four study days.

Randomization will be undertaken by a third party unconnected with the study using the randomization generator available at the www.randomization.com website. Randomisation will be generated for 16 sequential volunteers, and each random sequence generated will be placed in order in a sequentially numbered opaque, sealed envelope (1-16). As each volunteer is recruited onto the study, the appropriately numbered envelope will be opened by the study scientist to reveal the assignment to the group A or B. Note the study is single-blinded so the participants will be unaware to which group they will be allocated and which test meal they will be receiving on each experimental day.

8.1. Visit 3: First experimental day

After recording all food and drink consumed on the day before the first experimental day (pre-test diet) in a food diary provided by the study team (Annex 12), volunteers will undergo a 10 hour overnight fast from 10:00pm and attend the CRTU at approximately 8:00 am the following morning. During the fasting period volunteers will be advised to drink water. On arrival in the CRTU a trained nurse and a scientist from the study team will be on hand to ensure that the volunteers are comfortable and relaxed. Female volunteers will also be asked whether they are menstruating and this will be noted by the study scientist. All clinical procedures described in the subsequent sections will be carried out by a fully trained nurse in the CRTU.

Prior to cannulation a CRTU nurse will take and record the volunteer's blood pressure. If the volunteer's blood pressure is <90/50 or <95/55 if symptomatic or >160/100 three further measurements may be taken and recorded at 5 minute intervals. If the blood pressure measurement remains outside these ranges after the third measurement, the volunteer will be excluded from the study, referred to their G.P. and the study day cancelled.

If the blood pressure is within these ranges a CRTU nurse will insert an intravenous (i.v.) cannula into an appropriate peripheral vein in one of the volunteer's arms. No more than two attempts will be made to insert the cannula on any one experimental day. The cannula will remain in situ for six hours. If there are any problems with the cannula during the 6 hour sampling period either the cannula will be removed and a second cannula inserted or, if there are no more than two blood samples remaining they may be taken by venepuncture. The cannula will be flushed with sterile normal saline between blood samples to maintain patency of the cannula.

After the cannula has been appropriately sited and the first blood sample taken, the volunteers from group A and B will receive:

- Test meal 1 for all volunteers (group A and B): 200ml cola jelly prepared from 200ml cola (Coca-Cola), 10ml of commercially available

Diet Cola flavoured Drink Concentrate (Sodastream, Lakeland) and gelatine (from animal source, Dr. Oetker) with the addition of ferrous gluconate (approx. 20mg +/- 2mg) in alginate beads (approx. 22.2g) followed by 200ml of diet cola drink (Coca Cola) with 3 placebo capsules (filled with 50mg commercially available dextrose acting as placebos for calcium and an iron capsules.)

Results of previous human intervention studies have shown that consumption of a cola drink has no effect on iron absorption and study participants find it a more palatable way of receiving iron doses in solution[27]. During the six hour cannulation period volunteers will have access to a drinking water dispenser (Aquarius, UK).

Blood samples (5ml each time) will then be taken at t=0 (at baseline), then after consumption of the test meal at: 20, 40, 60, 80, 100, 120, 150, 180, 240, 300 and 360 min after the ingestion of the oral doses and collected into appropriate tubes for the preparation of serum. Blood samples will subsequently be analysed for serum iron concentrations. During the day, any adverse events and all required information will be recorded by the study scientist on the experimental day record sheet (Annex 13) and dealt with in accordance with Good Clinical Practice and NRES guidelines.

After the final blood sample has been collected, volunteers will be offered and encouraged to consume lunch which they selected from the menu during the screening visit. Lunch will be prepared by Catering Direct, University of East Anglia and will consist of: a sandwich filled with ham, cheese or tuna mayonnaise, 1 piece of fresh fruit, 1 cake bar, 1 packet of crisps, and a fruit juice drink. Selected food will be picked up from the Zest food outlet located in the main square at the University of East Anglia just before completion of a study day.

Following a minimum seven day “washout” period, the volunteers will be asked to resume their one day pre-test diet, prior to the second experimental

day (Visit 4) i.e. there will be a minimum of one week between each experimental day.

8.2. Visit 4: second experimental day

For volunteers from group B this will be the final study day. Volunteers from group A will attend 2 more study days. Study participants will return to the CRTU for the second experimental day after a 10 hour overnight fast and they will receive the second test meal which will consist of:

- Group A: 200ml cola jelly prepared from 200ml cola (Coca-Cola), 10ml of commercially available Diet Cola flavoured Drink Concentrate (Sodastream, Lakeland) and gelatine (Dr. Oetker) with the addition of ferrous gluconate in alginate beads (approx. 22.2g) followed by 200ml of diet cola drink (Coca Cola) with 2 calcium phosphate capsules (300mg of calcium in each caplsue, in total 600mg of calcium) and one placebo capsule (filled with 50mg commercially available dextrose) in place of the iron capsule.
- Group B: 200ml cola jelly (prepared from 200ml cola (Coca-Cola), 10ml of commercially available Diet Cola flavoured Drink Concentrate (Sodastream, Lakeland) and gelatine (Dr. Oetker)), 1 ferrous gluconate capsule (prepared exclusively for each volunteer to match the iron content in the alginate beads administered in test meal 1) followed by two placebo capsules and 200ml of diet cola drink.

Blood sampling will take place in an identical fashion to the first experimental day and each sample assayed for serum iron concentration as before. Again, data will be collected with respect to any adverse events and reported accordingly (Annex 13). Once blood collection has been completed, volunteers will be offered and encouraged to consume a meal as on the first experimental day.

8.3. Visit 5: third experimental day

After a minimum washout period of 7 days, and following a 10 hour overnight fast, study participants from group A only will return to the CRTU for the third experimental day. On this day they will receive the following meal:

- 200ml cola jelly (prepared as above), without iron containing alginate beads, 2 calcium capsules (as above) and 1 ferrous gluconate capsule (prepared exclusively for each volunteer to match the iron content in the alginate beads administered in test meal 2 followed by 200ml of diet cola drink.

Blood sampling will take place in an identical fashion to the previous experimental days and will be assayed for serum iron concentration. Again, data will be collected with respect to any adverse events (Annex 13) and a meal will be offered at the end of the study day.

8.4. Visit 6: fourth experimental day

After a minimum washout period of 7 days, and following a 10 hour overnight fast, only study participants from group A will return to the CRTU for their final experimental day. They will receive the meal which group B received on the 2nd experimental day:

- 200ml cola jelly (prepared as above), 1 ferrous gluconate capsule (prepared exclusively for each volunteer to match iron content in the alginate beads administered in test meal 1) followed by two placebo capsules and 200ml of diet cola drink.

Blood sampling will take place in an identical fashion to the previous experimental days and will be assayed for serum iron concentration. Again, data will be collected with respect to any adverse events. (Annex 13) and a meal will be offered at the end of the study day.

9. Iron Dose Preparation

For each test meal an oral dose of iron (approximately 20mg), sufficient to perturb the normal serum iron concentration[2], will be given to apparently healthy adult volunteers after 10 hour (overnight) fast.

9.1. Ferrous gluconate preparation

Food grade ferrous gluconate powder (Cat.No. 1.03868.5000; VWR International LTD, UK) will be purchased by the project collaborator, Institute of Food research (IFR). Approximately 20mg (amount tailored to each volunteer following test meal 1, but should be in the range 18-22mg based on previous experience of preparing iron-loaded alginate beads) of iron as ferrous gluconate will be weighed on food grade scales located in the Human Nutrition Unit kitchen at IFR into empty gel colour capsules (from http://www.distinctivemedical.com/empty-gel-color-capsule-0-14013) by a study scientist who holds a level 2 award in food safety in catering. Ferrous gluconate capsules will be prepared the day before consumption by the volunteers.

9.2. Preparation of iron containing alginate beads

Iron containing alginate beads will be prepared by either the study scientist or a scientist from Institute of Food research, Natalia Perez-Moral (holds Food Hygiene Certificate) in the kitchen of the Human Nutrition Unit (HNU) where the specially purchased enacpsulator designated to prepare beads for human consumption only has been placed.

Alginate (sodium alginate, Ref. 3712-47, Danisco, DK) solution will be prepared by dissolving alginate in water (tap distilled in HNU kitchen) for 20 hours) to obtain 0.5% alginate (w/v) concentration. Once dissolved, the solution will be put through the encapsulator in order to shape the beads which will be collected into an iron gluconate and calcium chloride (Cat. No. 1.72570.1000; VWR International LTD, UK) solution at 50/50 ratio, to be loaded with iron for 20 hours. Once iron loading has been completed, the beads will be filtered for 5 minutes and after that they will be placed in a food grade container and stored in an HNU fridge (at 4ºC). The beads will be

collected by the study scientist and transported to Clinical Research Trial Unit ready to be used in an experiment on the same day.

A sample from each batch of beads will be analysed for the exact content of ferrous gluconate. The concentrations measured will be recorded next to the volunteer ID number (annex 13) in order to use exactly the same amount of iron when preparing the capsules filled with iron gluconate.

10. Test meal preparation and consumption

Preparation of cola jelly will be performed in CRTU kitchen the day before each study day. Gelatine will be dissolved in heated in microwave cola mixed with cola concentrate. Once slightly cooled, 200ml of the liquid jelly will be poured into a disposable plastic cup and placed in the fridge to set.

Next morning, on the experimental day plastic cups will be taken out of the fridge and approximately 22.2g (around 6 tea spoons) of iron containing alginate beads (depending on the test meal) will be stirred into the jelly in order to mask the presence of the beads, or if test meal is without alginate beads, the jelly will be just stirred to look similar to the jelly containing the beads. In addition to the jelly, participants will be given 200ml cola drink (poured directly form unopened 0.5L Coca-Cola bottle) poured into disposable plastic cup followed by 3 capsules (placebo or/and calcium or/and iron capsule). All preparation of the above test meals will take place in the CRTU kitchen, and subsequently put onto a tray and taken out to the volunteers.

11. Analytical methods

11.1. Serum sample preparation

Following collection into trace element and EDTA free tubes (BD, USA or Vacuette, Greiner, UK depending on availability) blood samples will be allowed to stand at room temperature for a period of 30-60minutes prior to centrifugation at 1500g. The serum (approx. 2ml supernatant) will be removed and aliquoted into screw-top eppendorfs and stored at -80°C prior to analysis.

11.2. Serum sample analysis

Total serum iron (with no need for any pre-treatment) will be measured in duplicate using the QuantichromTM iron assay kit (a colorimetric assay for the determination of iron at 590nm). The method in the above assay utilises chromogen that forms a blue coloured complex explicitly with iron (II). In order to measure iron (III) in the sample, during the assay procedure Fe^{3+} is reduced to Fe^{2+} thus allowing measurement of total iron concentration.

In addition, ferritin (using sprcroferritin, Ramco USA) and serum transferrin receptor (Ramco, USA) elisa assays will be performed using baseline blood samples to calculate body iron in order to fully characterise the iron status of the study group and to investigate reliability and fluctuation in body iron measurements.

Furthermore CRP assessment from baseline blood samples will be performed on each experimental day to establish if serum iron appearance could be affected by inflammation. This analysis will be performed at an accredited pathology laboratory at the NNUH, Norwich.

12. Confidentiality

Personal data will be handled in line with the regulations of the Data Protection Act 1998. Once recruited onto the study, volunteers will be assigned a code number with only the named study scientists approved by the Ethics Committee having access to allow them to link codes to volunteers. All personal data and biological samples will be coded with this number to ensure confidentiality. All personal information will be kept confidential and the information linking codes to participants will have restricted access known only to the chief investigator, academic supervisors, CRTU research nurses, and the volunteer's GP. It will not be possible for an individual to be identified solely from their code number.

12.1. Storage of the data

Electronic data stored within the UEA will be account password protected. Electronic data transferred via email is encrypted by the UEA web server. Only study scientists and appointed nurses will have access to the coded

information. It is expected that up to 50 potential volunteers will be screened in order to recruit a total of 16 volunteers successfully completing the study.

Professor Susan Fairweather-Tait, academic supervisor will act as custodian of the data generated in this study.

A hard copy of the datasheet linking names and addresses with code numbers will be kept in a locked filing cabinet at UEA MED with access restricted to the academic supervisor Professor Susan Fairewather-Tait and chief investigator Anna Wawer. Anonymised participant data will be stored on the secure network at UEA and CRTU and will not be stored on any external drives (including desk top C drives, laptops or external storage devices). Access to the files will only accessible to the staff associated with this project. Manual files containing the source data will be kept within the CRTU in locked storage cabinets during the intervention and once the intervention will be completed, the data will be stored in locked archive storage, located at the MED building at UEA for 5 years after the completion of the study. In addition, coded 'non-identifiable' patient data will be kept in secure storage, with access limited to study staff, and the information will be archived in locked file cabinets for 5 years and then destroyed appropriately. Biological samples will be stored as cell-free extracts for analysis and retained at the study sites to be accessed by study staff for 5 years from collection and then destroyed using standard biohazard procedures in place at the Biomedical Research Centre at the UEA.

13. Volunteers expenses / payments

For taking part in the study participants will receive the following inconvenience payments:

- Volunteer travel costs up to £10 for each visit to the CRTU *Note: Travel expenses will be reimbursed on production of a receipt for public transport or at 40p per mile rate for private cars.*

- In addition volunteers will be paid £85 for each experimental day (£65 for cannulation, £10 for keeping a food diary and £10 for consuming a test meal) at the end of the study (Annex 14a & 14b).

If the volunteer withdraws from the study before completion inconvenience payments will be paid pro rata.

14. Statistical analysis

Based on previous similar studies the standard deviation of iron absorption is expected to be approximately 3%. Since absorption is related to iron stores it would be expected that there will be a good correlation between iron absorption from the iron loaded alginate beads and ferrous gluconate in its native form i.e. volunteers who absorb higher amounts of one form will also absorb higher amounts of the other form.

14.1. Statistical power

Each volunteer will act as his/her own control, which will minimise the number of subjects required. The assumptions made for the power calculation to estimate the number of volunteers are that a difference of 2.5% in iron absorption between the 2 tests (primary outcome, test meal 1 and test meal 4) will be nutritionally significant. A minimum of 12 volunteers would be required to complete the study to detect a difference of 2.5% at a significance level of 0.05 for 80% power.

For the secondary aim, a minimum of 8 volunteers would be required to detect a significant difference in iron absorption of 3.3% between a test meal containing 20mg of iron as iron gluconate with calcium and a test meal containing 20mg of iron as iron gluconate with calcium and alginate beads (test meals 2 and 3). This assumes a power of 80%, a level of significance of 0.05 and standard deviation of differences (within pairs) of 2.8%

14.2. Statistical method

The primary outcome measure is the difference in iron (ferrous gluconate) absorption when consumed in a form of iron incorporated into alginate beads compared with the same amount of iron in an unprotected form i.e. not in alginate beads. The secondary outcome measure is the difference in iron

(ferrous gluconate) absorption when consumed in presence of calcium in a form of iron incorporated into alginate beads compared with the same amount of iron not in alginate beads in presence of calcium.

Paired, two-tailed Student's t test will be used to assess any significant differences in absorption (for the primary and secondary outcome measure). This method was found to be appropriate when assessing iron absorption in previous studies conducted at the Institute of Food Research[4].

14.3. Data analysis

Dainty et al[4] have previously shown that the measurement of serum iron levels for 6 hours after an oral dose is a simple and effective method for estimating iron absorption which correlates well with the more complicated and expensive measure of absorption using stable isotopes of iron. From the serum iron accumulation, it will be possible to calculate percentage iron absorption from the administered dose using the following method:

Given a time of between 3 and 6 hours post administration of an oral dose ($dose_{oral}$) the rate of infusion R can be calculated from the serum iron concentration

$$R = \frac{\text{mass of dose that is absorbed}}{\text{time period for absorption}} = \frac{M}{T} \qquad (1)$$

Given a volume of distribution V, and a rate of elimination from the compartment k, the concentration in the compartment can be approximated to:

$$C = \frac{M}{V.T.k} \cdot (1 - e^{-k.t}) \quad (0 < t < T) \qquad (2)$$

$$C = \frac{M}{V.T.k} \cdot (1 - e^{-k.T})\,.e^{-k(t-T)} \quad (T < t) \qquad (3)$$

By fitting the equations to the concentration data, M, T and k can be calculated.

The fractional absorption from the oral dose is then:

$$\text{Fractional absorption} = \frac{\mathrm{M}}{\text{dose}_{\text{oral}}}. \qquad (4)$$

15. References:

1. Villalpando, S. et al. Fortifying milk with ferrous gluconate and zinc oxide in a public nutrition program reduced the prevalence of anemia in toddlers. *The Journal of nutrition* **136**, 2633-7(2006).

2. Conway, R.E. et al. Serum Iron Curves Can Be Used to Estimate Dietary Iron Bioavailability in Humans. *The Journal of nutrition* 1910-1914(2006).

3. Cook, J.D., Dassenko, S. a & Whittaker, P. Calcium supplementation: effect on iron absorption. *The American journal of clinical nutrition* **53**, 106-11(1991).

4. Dainty, J.R. et al. Quantification of unlabelled non-haem iron absorption in human subjects: a pilot study. *British Journal of Nutrition* **90**, 503(2003).

5. Sarria, B. et al. Estimation of iron absorption in humans using compartmental modelling. *European journal of clinical nutrition* **59**, 142-4(2005).

6. Cook, J.D. et al. Serum ferritin as a measure of iron stores in normal subjects. *The American journal of clinical nutritionAmerican Journal of Clinical Nutrition* 681 -687(1974).

7. Harvey, L.J. et al. Impact of menstrual blood loss and diet on iron deficiency among women in the UK. *British Journal of Nutrition* **94**, 557(2005).

8. Zimmermann, M.B. & Hurrell, R.F. Nutritional iron deficiency. *Lancet* **370**, 511-20(2007).

9. McLean, E. et al. Worldwide prevalence of anaemia, WHO Vitamin and Mineral Nutrition Information System, 1993-2005. *Public health nutrition* **12**, 444-54(2009).

10. Peter Aggett, Ann Prentice, Philip Calder, Sue Fariweather-Tait, Sally Grantham-Mc Gregor, Christine Gratus, Timothy Key, Joe Lunec, Kim Fleischer Michaelsen, Martin Pippard, M.W. Iron and health. Scientific Advisory Committee on Nutrition. (2010).

11. Hunt, J.R. Bioavailability of iron, zinc, and other trace minerals from vegetarian diets. *The American journal of clinical nutrition* **78**, 633S-639S(2003).

12. Derbyshire, E. et al. Iron deficiency - is there a role for the food industry? *International Journal of Food Science & Technology* **45**, 2443-2448(2010).

13. Hurrell, R.F. Fortification : Overcoming Technical and Practical Barriers. *The Journal of nutrition* **132**, 806-812(2002).

14. J, F., JM, F. & T, O. One hundred and twenty-seven cultured human tumor cell lines producing tumors in nude mice. *journal of national cancer institute* **59**, 221-226(1997).

15. Hidalgo IJ, Raub TJ, B.R. Characterization of the human colon carcinoma cell line (Caco-2) as a model system for intestinal epithelial permeability. *Gastroenterology* **96**, 736-749(1989).

16. Florence Delie, W.R. A Human Colonic Cell Line Sharing Similarities With Enterocytes as a Model to Examine Oral Absorption: Advantages and Limitations of the Caco-2 Model. *Critical reviews in Therapeutic Drug Carrier Systems.* **14**, 221-286(1997).

17. Quaroni, A. et al. Epithelioid cell cultures from rat small intestine. Characterization by Morphologic and Immunologic Criteria. *Journal of Cell biology* **80**, 248-265(1979).

18. Glahn, R.P. et al. Caco-2 Cell Iron uptake from Meat and Casein Digests Parallels In Vivo Studies : use of a Novel In Vitro Method for Rapid Estimation of Iron Bioavailability. *The Journal of nutrition* **126**, 332-339(1996).

19. Glahn, R.P. et al. Caco-2 Cell Ferritin Formation Predicts Nonradiolabeled Food Iron Availability in an In Vitro Digestion / Caco-2 Cell Culture Model. *The Journal of nutrition* **128**, 1555-1561(1998).

20. Fairweather-Tait S, Lynch S, Hotz C, Hurrell R, Abrahamse L, Beebe S, Bering S, Bukhave K, Glahn R, Hambidge M, Hunt J, Lonnerdal B, Miller D, Mohktar N, Nestel P, Reddy M, Sandber AS, Sharp P, Teucher B, T.T. The usefulness of in vitro models to predict the bioavailability of iron and zinc: a consensus statement from the HarvestPlus expert consultation. *International Journal for Vitamin and Nutrition Research* **75**, 371-374

21. Au, a P. & Reddy, M.B. Caco-2 cells can be used to assess human iron bioavailability from a semipurified meal. *The Journal of nutrition* **130**, 1329-34(2000).

22. Larsen, B. & Haug, A. Biosynthesis of alginate. *Carbohydrate research* 287-296(1970).

23. Emerton, V. & Choi, E. *Essential Guide to Food Additives*. 182-187(2008).

24. Chan, L., Jin, Y. & Heng, P. Cross-linking mechanisms of calcium and zinc in production of alginate microspheres. *International journal of pharmaceutics* **242**, 255-8(2002).

25. Sreeram, K. Studies on the nature of interaction of iron(III) with alginates. *Biochimica et Biophysica Acta (BBA) - General Subjects* **1670**, 121-125(2004).

26. Sandberg, A.-sofie et al. Alginate, small bowel sterol excretion, and absorption of nutrients in ileostomy subjects. *The American journal of clinical nutrition* 751-6(1994).

27. Collings, R. et al. Low-pH Cola Beverages Do Not Affect Women ' s Iron Absorption from a Vegetarian Meal. *The Journal of nutrition* **141**, 805-808(2011).

List of appendixes

Appendix 1. *In vitro* results

Alginates D1 and D4

Both alginates (D1 and D4) at 1% (w/v) concentrations in the presence of iron significantly increased ferritin expression in comparison with positive control samples (*p* =0.001 and 0.007 respectively). In two cases where alginate D1 was used at concentrations of 0.5% and 1% (w/v) in the presence of ascorbic acid and iron, the levels of ferritin formation were significantly higher (p<0.001 and 0.0120 respectively) than in positive controls in the presence of ascorbic acid.

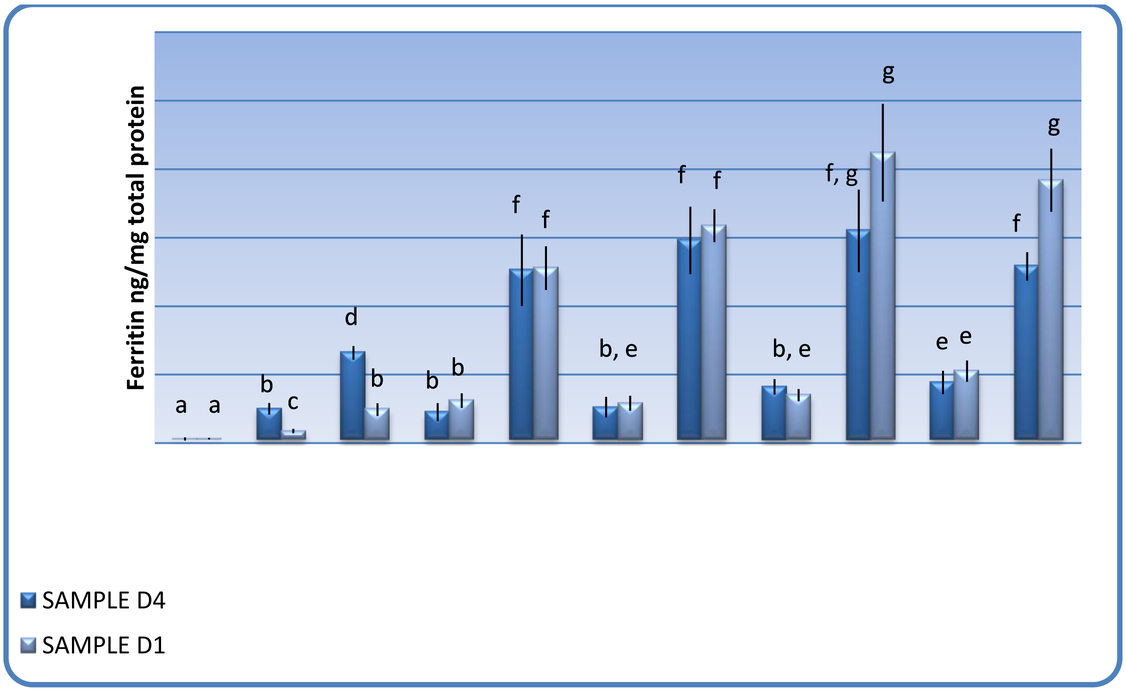

Figure A. Ferritin formation in Caco-2 cells treated with D1 and D4 alginates with and without Ferric Ammonium Citrate (FAC) and Ascorbic Acid (AA). Data represents mean±SD (n=4 except FAC and FAC +AA, n=3). Letters denote significantly different groups.

Alginates Manucol and Manugel

Concentrations of 0.5% and 1% (w/v) of Manucol or Manugel alginates in presence of iron significantly increased ferritin formation in comparison with samples treated with iron only (*p* <0.001).

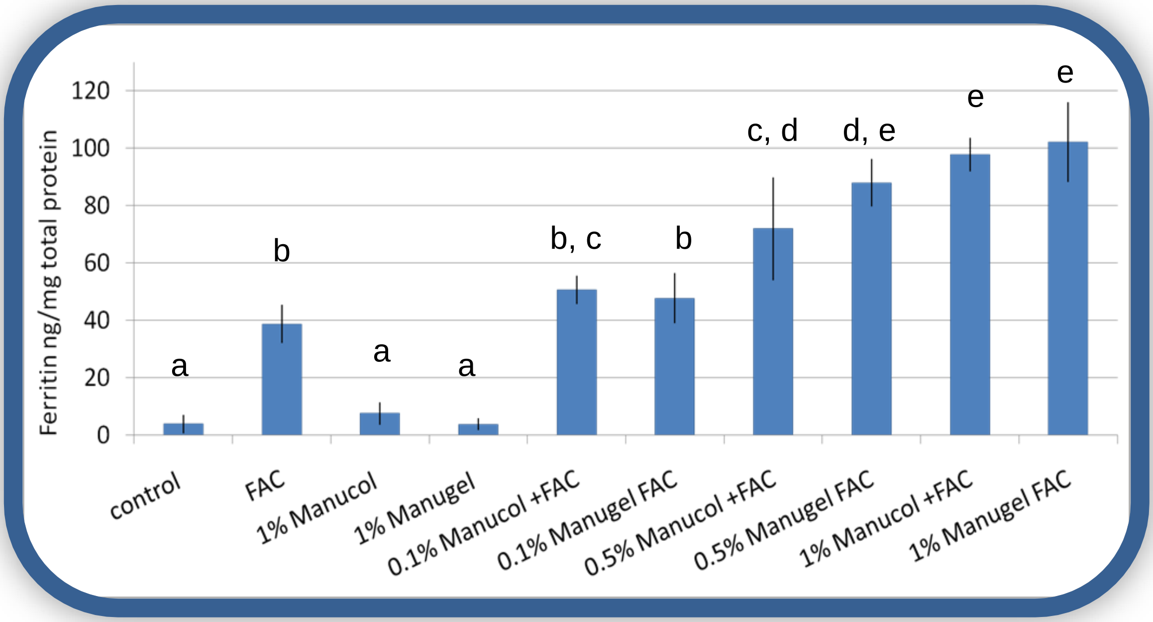

Figure B. Ferritin formation in Caco-2 cells treated with Manucol and Manugel alginates with and without FAC. Data represents mean±SD (n=4, except control and FAC n=6). Letters denote significantly different groups.

Iron loaded alginate beads

There was no significant difference between samples treated with iron in the presence of ascorbic acid in comparison with samples treated with ferrous gluconate present in alginate beads. Data obtained revealed a significant increase in ferritin formation in all samples treated with ferrous gluconate loaded beads (FeG1, FeG2, FeG3) in comparison with control samples (p=0.005*;* p=0.001 *and* p=0.019 *respectively).* However, this was not the case for beads loaded with FAC. There was no significant effect of increasing content of alginate in relation to quantity of iron used.

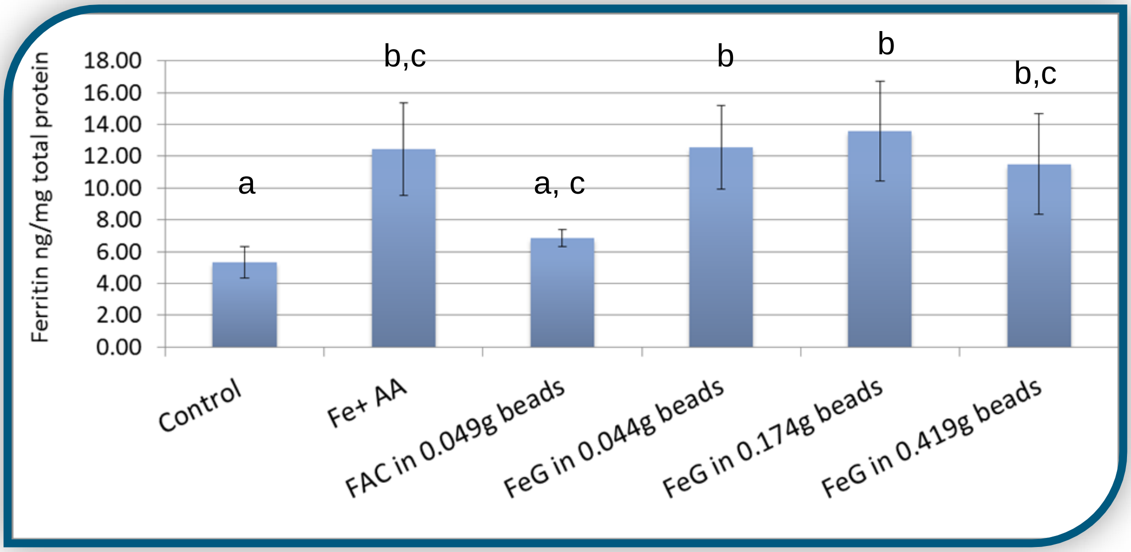

Figure C. Ferritin formation in Caco-2 cells treated with iron loaded alginate beads. Data represents mean±SD (n=4), letters denote significantly different groups.
