# Supplementary material for: Alginate Inhibits Iron Absorption from Ferrous Gluconate in a Randomized Controlled Trial and Reduces Iron Uptake into Caco-2 Cells
Source: PLoS One. 2014 Nov 12;9(11):e112144. doi: 10.1371/journal.pone.0112144 (PMC4229116; doi:10.1371/journal.pone.0112144)
Supplement: Checklist S1 — CONSORT checklist. (DOCX) [file pone.0112144.s002.docx]

| **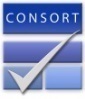** | **CONSORT 2010 checklist of information to include when reporting a randomised trial*** | | |
| --- | --- | --- | --- |
| **Section/Topic** | **Item**  **No** | **Checklist item** | **Reported**  **on page**  **No** |
| Title and  abstract |  |  |  |
|  | 1a | Identification as a randomised  trial in the title | Title |
|  | 1b | Structured summary of trial  design, methods, results, and  conclusions (for specific guidance  see CONSORT for abstracts) | Abstract |
| Introduction |  |  |  |
| Background  and objectives | 2a | Scientific background and  explanation of rationale | Introduction |
|  | 2b | Specific objectives or hypotheses | Introduction |
| Methods |  |  |  |
| Trial design | 3a | Description of trial design (such  as parallel, factorial) including  allocation ratio | Materials and  Methods, Human  study |
|  | 3b | Important changes to methods  after trial commencement (such  as eligibility criteria), with reasons | n/a |
| Participants | 4a | Eligibility criteria for participants | Materials and  Methods, Human  study |
|  | 4b | Settings and locations where the  data were collected | Materials and  Methods, Human  study |
| Interventions | 5 | The interventions for each group  with sufficient details to allow  replication, including how and  when they were actually  administered | Materials and  Methods, Human  study |
| Outcomes | 6a | Completely defined pre specified  primary and secondary outcome  measures, including how and  when they were assessed | Materials and  Methods, Human  study |
|  | 6b | Any changes to trial outcomes  after the trial commenced, with  reasons | n/a |
| Sample size | 7a | How sample size was determined | Materials and  Methods, Statistical  Methods |
|  | 7b | When applicable, explanation of  any interim analyses and  stopping guidelines | n/a |
| Randomisation: |  |  |  |
| Sequence  generation | 8a | Method used to generate the  random allocation sequence | Materials and  Methods, Human  study |
|  | 8b | Type of randomisation; details of  any restriction such as blocking  and block size) | n/a |
| Allocation  Concealment  mechanism | 9 | Mechanism used to implement  the random allocation sequence  (such as sequentially numbered  containers), describing any steps  taken to conceal the sequence  until interventions were assigned | Materials and  Methods, Human  study |
| Implementation | 10 | Who generated the random  allocation sequence, who  enrolled participants, and who  assigned participants to  interventions | Materials and  Methods, Human  study |
| Blinding | 11a | If done, who was blinded  after assignment to  interventions (for example,  participants, care providers,  those assessing outcomes)  and how | Materials and  Methods, Human  study |
|  | 11b | If relevant, description of the  similarity of interventions | Materials and  Methods, Human  study |
| Statistical  methods | 12a | Statistical methods used to  compare groups for primary  and secondary outcomes | Materials and  Methods, Statistical  Methods |
|  | 12b | Methods for additional analyses,  such as subgroup analyses and  adjusted analyses | Materials and  Methods, Statistical  Methods |
| Results |  |  |  |
| Participant flow  (a diagram is  Strongly  recommended) | 13a | For each group, the numbers of  participants who were randomly  assigned, received intended  treatment, and were analysed for  the primary outcome | Figure 1 |
|  | 13b | For each group, losses and  exclusions after randomisation,  together with reasons | Figure 1 |
| Recruitment | 14a | Dates defining the periods  of recruitment and follow-up | Materials and  Methods, Human  study |
|  | 14b | Why the trial ended or was  stopped | n/a |
| Baseline data | 15 | A table showing baseline  demographic and clinical  characteristics for each  group | Supporting  Information  file |
| Numbers  analysed | 16 | For each group, number of  participants (denominator)  included in each analysis and  whether the analysis was by  original assigned groups | Figure 1 |
| Outcomes and  estimation | 17a | For each primary and secondary  outcome, results for each group,  and the estimated effect size and  its precision (such as 95%  confidence interval) | Materials and  Methods, Human  study, 13-14,  Table 1 |
|  | 17b | For binary outcomes,  presentation of both absolute and  relative effect sizes is  recommended | Materials and  Methods, Statistical  Methods; Results |
| Ancillary  analyses | 18 | Results of any other analyses  performed, including subgroup  analyses and adjusted analyses,  distinguishing pre-specified from  exploratory | Materials and  Methods, Statistical  Methods; Results |
| Harms | 19 | All important harms or  unintended effects in each  group (for specific guidance  see CONSORT for harms) | n/a |
| Discussion |  |  |  |
| Limitations | 20 | Trial limitations, addressing  sources of potential bias,  imprecision, and, if relevant,  multiplicity of analyses | Results; Discussion |
| Generalisability | 21 | Generalisability (external validity,  applicability) of the trial findings | Discussion |
| Interpretation | 22 | Interpretation consistent with  results, balancing benefits and  harms, and considering other  relevant evidence | Results; Discussion |
| Other |  |  |  |
| Registration | 23 | Registration number and name of  trial registry | Title page;  Materials and  Methods, Human  study |
| Protocol | 24 | Where the full trial protocol can  be accessed, if available | Materials and  Methods, Human  study |
| Funding | 25 | Sources of funding and other  support (such as supply of drugs) | Journal submission |
