# Supplementary material for: Alginate Inhibits Iron Absorption from Ferrous Gluconate in a Randomized Controlled Trial and Reduces Iron Uptake into Caco-2 Cells
Source: PLoS One. 2014 Nov 12;9(11):e112144. doi: 10.1371/journal.pone.0112144 (PMC4229116; doi:10.1371/journal.pone.0112144)
Supplement: Table S2 — Individual data for ferrous gluconate absorption with and without alginate. (DOCX) [file pone.0112144.s004.docx]

**Table S2.** **Individual data for absorption of ferrous gluconate (% of dose absorbed) with and without alginate beads**

|  | % of iron dose absorbed | |
| --- | --- | --- |
|  | Test meal 1 | Test meal 2 |
| Recoded volunteer number | Fe in alginate beads | Fe alone |
| ALG1 | 5.4 | 14.2 |
| ALG2 | 8.2 | 15.7 |
| ALG3 | 12.7 | 16.1 |
| ALG4 | 6.3 | 13.1 |
| ALG5 | 6.6 | 15.7 |
| ALG6 | Missing data | 16 |
| ALG7 | 6.3 | 11.9 |
| ALG8 | 5.7 | 7.5 |
| ALG9 | 8.4 | 8.7 |
| ALG10 | Missing data | 5.7 |
| ALG11 | 10.5 | 6.3 |
| ALG12 | 8.1 | 8.8 |
| ALG13 | 4.6 | 11.4 |
| ALG14 | 14.6 | 16.4 |
| ALG15 | 13.0 | 17.9 |
| *Mean (SD) % absorption for complete pairs (n=13)  Median, range (n=13)  95%CI (n=13) | 8.5^◊^(3.2)  8.1; (4.6-14.6)  (6.7,10.2) | 12.6^∆^(4.1)  13.1 (5.7-17.9)  (10.5,14.6) |

*Means without a common symbol are significantly different within the pairs compared
