# Supplementary material for: Alginate Inhibits Iron Absorption from Ferrous Gluconate in a Randomized Controlled Trial and Reduces Iron Uptake into Caco-2 Cells
Source: PLoS One. 2014 Nov 12;9(11):e112144. doi: 10.1371/journal.pone.0112144 (PMC4229116; doi:10.1371/journal.pone.0112144)
Supplement: Table S3 — Individual data for ferrous gluconate absorption with and without alginate and calcium. (DOCX) [file pone.0112144.s005.docx]

**Table S3. Individual data for absorption of ferrous gluconate (% of dose absorbed) with and without alginate and calcium (Ca)**

|  | % of iron dose absorbed | | | |
| --- | --- | --- | --- | --- |
|  | Test meal 1 | Test meal 2 | Test meal 3 | Test meal 4 |
| Recoded volunteer number | Fe in alginate beads | Fe | Fe in alginate beads + Ca | Fe + Ca |
| ALG7 | 6.3 | 11.9 | 3.5 | 4.9 |
| ALG8 | 5.7 | 7.5 | 5.4 | - |
| ALG9 | 8.4 | 8.7 | 3.2 | 5.3 |
| ALG10 | - | 5.7 | - | 5.6 |
| ALG11 | 10.5 | 6.3 | 6.1 | - |
| ALG12 | 8.1 | 8.8 | 4.8 | 6.3 |
| ALG13 | 4.6 | 11.4 | 4.4 | 6.3 |
| ALG14 | 14.6 | 16.4 | 9.3 | 6.6 |
| ALG15 | 13.0 | 17.9 | - | 4.2 |
| Mean (SD) %  absorption for complete pairs across:  *Test meal 1 and 3 (n=7)  *Test meal 2 and 4 (n=7)  Median; range (n=7)  95% CI (n=7) | 8.3^◊^ (3.4)  -  8.1; (4.6-14.6)  (5.8,10.8) | -  11.5^1^ (4.3)  11.4; (5.7-17.9)  (8.3,14.8) | 5.2^∆^ (2.0)  -  4.8; (3.2-9.3) (3.7,6.8) | -  5.6^2^ (0.9)  5.6 (4.2-6.6)  (5.0,6.3) |
| Mean (SD) % absorption for complete pairs (all 4 tests; n=5)*  Median; range (n=5)  95% CI (n=5) | 8.4^a^(3.8)  8.1; (4.6-14.6)  (5.1,11.7) | 11.4^b^(3.1)  13.1; (5.7-17.9)  (8.7-14.2) | 5.0^c^(2.5)  4.8; (3.2-9.3)  (2.9,7.2) | 5.9^a, c^(0.7)  5.6; (4.2-6.6)  (5.2,6.5) |

*Means without common symbol, number or letter are significantly different within the pairs compared
