# Supplementary material for: Alginate Inhibits Iron Absorption from Ferrous Gluconate in a Randomized Controlled Trial and Reduces Iron Uptake into Caco-2 Cells
Source: PLoS One. 2014 Nov 12;9(11):e112144. doi: 10.1371/journal.pone.0112144 (PMC4229116; doi:10.1371/journal.pone.0112144)
Supplement: Form S1 — Consent form. (DOC) [file pone.0112144.s006.doc]

Volunteer identification number for this trial:

**CONSENT FORM**

**Version 1 (26th August 2011)**

**Title of Project: Pilot study to measure the absorption of iron from ferrous gluconate incorporated into alginate beads.**

Name of Researchers: Professor Susan Fairweather-Tait, Ms Anna Wawer

**Please initial box**

⬜

1. I confirm that I have read and understand the information sheet dated (version ) for the

above study. I have had the opportunity to consider the information, ask questions and

have had these answered satisfactory.

⬜

1. I understand that my participation is voluntary and that I am free to withdraw at any time,

without giving any reason, without my medical care or legal rights being affected.

1. I understand that data collected during the study may be looked at by individuals

⬜

from University of East Anglia, from regulatory authorities or from the NHS Trust,

where it is relevant to my taking part in this research. I give permission for these

individuals to have access to my records.

⬜

1. I agree for my GP to be informed about my taking part in the above study.

⬜

5. I agree to take part in the above study.

_________________________ _______________________ _______________

Name of Participant Signature Date

_________________________ ______________________ ________________

Name of Person taking consent Signature Date

(if different from researcher)

_________________________ _______________________ ________________

Researcher Signature Date

1 for participant; 1 for researcher site file

Page 1 of 1
