# Supplementary material for: Alginate Inhibits Iron Absorption from Ferrous Gluconate in a Randomized Controlled Trial and Reduces Iron Uptake into Caco-2 Cells
Source: PLoS One. 2014 Nov 12;9(11):e112144. doi: 10.1371/journal.pone.0112144 (PMC4229116; doi:10.1371/journal.pone.0112144)
Supplement: Form S2 — Health questionnaire. (DOC) [file pone.0112144.s007.doc]

**CONFIDENTIAL**

**Volunteer Screening Questionnaire: Iron & Alginate Study**

Volunteer code number…………………… Sex:…………………….................

Date of birth:………………………………. Age:………………………………..

Height:……………………………………… Weight:…………………………….

Blood Pressure:…………………………… Body Mass Index (BMI)…………..

Have you ever had any of the following? If yes give details below each relevant section.

Angina/heart disease: Y N Thrombosis: Y N

…………………………………….. ………………………………………….

…………………………………….. ………………………………………….

High Blood Pressure: Y N High Cholesterol: Y N

……………………………………. …………………………………………..

……………………………………. …………………………………………..

Chest problems: Y N Diabetes: Y N

……………………………………. ……………………………………………

……………………………………. ……………………………………………

Gastrointestinal surgery: Y N Digestive problems: Y N

……………………………………. ……………………………………………

……………………………………. …………………………………………...

Blood disorders: Y N Other:

……………………………………. …………………………………………….

……………………………………. …………………………………………….

Are you currently on any the following:

**If yes, give details below each relevant section of brand, dosage, frequency, when started etc.**

Prescribed medication: Y N

………………………………………………………………………………………………………

………………………………………………………………………………………………………

………………………………………………………………………………………………………

Page 1 of 3

Volunteer code number:………………………………………..

Dietary Supplements: Y N iron tablets in the last 12 months: Y N

…………………………………………. ……………………………………………..

…………………………………………… ………………………………………………

…………………………………………… ………………………………………………

…………………………………………… ………………………………………………

Have you given blood in the last 3 months: Y N

If yes give details below:

…………………………………………………………………………………………………………..

…………………………………………………………………………………………………………..

…………………………………………………………………………………………………………..

Are you currently suffering from any illness/injury: Y N

If yes give details below:

………………………………………………………………………………………………………….

………………………………………………………………………………………………………….

…………………………………………………………………………………………………………..

Have you had a blood transfusion in the last 3 months: Y N

…………………………………………………………………………………………………………..

…………………………………………………………………………………………………………..

…………………………………………………………………………………………………………..

Do you smoke? Y N

**Note if you answered YES then we are unable to accept you on this study.**

Have you ever smoked: Y N

When did you stop smoking:………………….

Do you drink alcohol: Y N

If yes how many units per week:…………………………………………………………………..

Have you any known allergies: Y N

Food:………………………………………… Drugs:……………………………………………….

Other:………………………………………………………………………………………………….

…………………………………………………………………………………………………………..

Special dietary requirements: Y N

If yes state:……………………………………………………………………………………………

…………………………………………………………………………………………………………..

Page 2 of 3

Volunteer code number:………………………………………..

Do you agree to us informing your General Practitioner of your participation in the study or of any results found: Y N

**If you have answered NO to this question then we are unable to accept you on this study.**

Name and Address of your General Practitioner:

……………………………………………………………………………………………………………

……………………………………………………………………………………………………………

……………………………………………………………………………………………………………

Telephone number:……………………………….

Form completed by (print):……………………….. Signature:……………………………….

Date:………………………………………………….

Page 3 of 3
