# Supplementary material for: Alginate Inhibits Iron Absorption from Ferrous Gluconate in a Randomized Controlled Trial and Reduces Iron Uptake into Caco-2 Cells
Source: PLoS One. 2014 Nov 12;9(11):e112144. doi: 10.1371/journal.pone.0112144 (PMC4229116; doi:10.1371/journal.pone.0112144)
Supplement: Form S3 — Adverse events form. (DOC) [file pone.0112144.s008.doc]

**CONFIDENTIAL**

**ALGINATE & IRON STUDY: Side effect record sheet**

***Form to be completed by Study scientist or CRTU nurse***

Volunteer code number……………………

Date of birth:………………………………. Age:………………………………..

Date ……………………………….. Experimental Day*: Day 1 / Day 2 / Day 3 / Day 4

Test meal*: with alginate beads / without alginate beads / with iron capsule /without iron capsule /with calcium /without calcium

( *delete as appropriate)

Time dose administered: ………………………………………………………………………….

Time of last blood sampling: ……………………………………………………………………...

*Please record below details of tolerability or side effects reported by the study participant during the study day in response to the test doses*

| **TIME** | **DETAILS REPORTED** |
| --- | --- |
|  |  |

Person completing form: ……………………………………………………………………………

Signature: …………………………………… Date: ………………………………………….
