# Supplementary material for: Alginate Inhibits Iron Absorption from Ferrous Gluconate in a Randomized Controlled Trial and Reduces Iron Uptake into Caco-2 Cells
Source: PLoS One. 2014 Nov 12;9(11):e112144. doi: 10.1371/journal.pone.0112144 (PMC4229116; doi:10.1371/journal.pone.0112144)
Supplement: Form S4 — Subject record sheet. (DOCX) [file pone.0112144.s009.docx]

**IRON & ALGINATE STUDY RECORD SHEET**

VOLUNTEER ID:………………………………………

STUDY DAY*: day 1 day 2 day 3 day 4 ( *circle as appropriate)

DATE:……………………………………………………….

FEELING WELL*: ………………………………………Y N ( *circle as appropriate)

BLOOD PRESSURE (max. 3 measurements)..............................................................................................

……………………………………………………………...................................................................In range*…. Y N

Female volunteers: menstruating*……..……Y N

NO. of days since last day of end of menstruation:………….

Test meal*: with alginate beads / without alginate beads / with iron capsule /without iron capsule /with calcium /without calcium ( *circle as appropriate)

| BLOOD SAMPLE | Baseline blood sample | Meal | 20min blood sample | 40min blood sample | 60min blood sample | 80min blood sample |
| --- | --- | --- | --- | --- | --- | --- |
| COLLECTED Y/N |  | ---------- |  |  |  |  |
| TIME OF  COLLECTION |  | ______ |  |  |  |  |
| TIME OF MEAL ADMINISTRATION | ______ |  | _______ | ______ | ______ | ______ |

| BLOOD SAMPLE | 100min blood sample | 120min blood sample | 150min blood sample | 180min blood sample | 240min blood sample | 300min blood sample | 360min blood sample |
| --- | --- | --- | --- | --- | --- | --- | --- |
| COLLECTED Y/N |  |  |  |  |  |  |  |
| TIME OF COLLECTION |  |  |  |  |  |  |  |

FULL STUDY DAY COMPLETED………………………Y N (if no please refer to NOTES section)

CONSUMED LUNCH……………………………………….Y N

BEADS BATCH NUMBER:…………………………………

IRON CONTENT (mg as Fe)………………………………

**NOTES** (study scientist & participants comments)

| **TIME** | **DETAILS REPORTED** |
| --- | --- |
|  |  |

Person completing form: …………………………………

Signature: …………………………………… Date: ………………………………………….
