# Supplementary material for: Alginate Inhibits Iron Absorption from Ferrous Gluconate in a Randomized Controlled Trial and Reduces Iron Uptake into Caco-2 Cells
Source: PLoS One. 2014 Nov 12;9(11):e112144. doi: 10.1371/journal.pone.0112144 (PMC4229116; doi:10.1371/journal.pone.0112144)
Supplement: Form S5 — Research Ethics application. (PDF) [file pone.0112144.s010.pdf]

UNIVERSITY OF EAST ANGLIA
FACULTY OF HEALTH ETHICS COMMITTEE
Application Form for Ethical Approval of a Research Project

Please refer to the guidelines when completing this form.
This document should help members of the FOH Ethics Committee understand the objectives of your project/research and the procedures to be conducted.
It is ESSENTIAL that you use non-technical language that can easily be understood by non-specialists and lay members of the Committee and all applications need to include all relevant documents. It is not acceptable to refer the committee to a protocol, and the information on the application together with the attachments should be sufficient to allow the Committee to form an opinion. Forms may be reviewed by the Chair and will be returned to you if you do not meet these requirements. This will delay approval of your application as applications cannot be accepted after the deadline.

Does the project involve the use of ***drugs, or testing of new equipment****, or research on* ***NHS staff or patients****? If so, it* ***MUST*** *be referred to an NHS Research Ethics Committee for approval and the Faculty of Health Ethics Committee must be informed of the outcome.*

1. **Name of applicant:** ANNA WAWER
(Block letters)

2. **Academic address for correspondence:** *University of East Anglia, Norwich Medical School, Department of Nutrition, BMRC building, floor 0.*

Norwich, Norfolk …………..Post code: *NR4 7TJ*

3. **Tel No**: *07746362896* **Fax No**: *01603 593752*

4. **E-mail address:** *a.wawer@uea.ac.uk*

5. **School** (AHP, MED, NAM): MED ………………………………………………

6. **Status of applicant** (Staff, UG or PG student - and year of course)*: PG student, 3rd year*

7. **If Student:**
Is this study being carried out to fulfil a required part of your course? *Yes*

If No:
Please confirm contact details of supervisor

…………………………………………………………………………………………

Name of supervisor

8. Has this application gone to an Ethics Committee elsewhere? *NO*

If YES, please indicate where and include copies of correspondence:

……………………………………………………………………………………

Please send 16 copies of the proposal and application form (stapled together in the top left-hand corner) to: Maggie Rhodes, FOH Research Office, Elizabeth Fry Building Room 2.30, University of East Anglia, Norwich NR4 7TJ; plus an e-mail copy to margaret.rhodes@uea.ac.uk on or before the deadline shown on the website (http://www.uea.ac.uk/foh/research/ethics-committee).

For any queries telephone: Maggie Rhodes 01603 597190.

Project details (please could sections 9, 10 and 11 be limited to a maximum of 3000 words.

9. Full title: *Study to measure the absorption of iron from ferrous gluconate incorporated into alginate beads*………………………………………………

10. Purpose of project:

Iron deficiency anaemia affects around two billion people worldwide. Food fortification with iron is a realistic way to combat this problem. Water soluble forms of iron are considered to be more bioavailable than non-soluble iron compounds. However, the former often cause sensory problems when added to foods. As a potential strategy for overcoming this problem we will use alginate, in the form of alginate beads, as a water resistant barrier which may prevent potential organoleptic changes. It may also protect iron from oxidation during digestion and becoming unavailable for uptake into the body.
Once participants will be recruited, they will be asked to come in to Clinical Research and Trials Unit fasted on two or four occasions (depending to which group they will be randomly assigned). On each of the occasions trained nurse will insert an intravenous cannula into an appropriate peripheral vein in one of the volunteer's arms. The cannula will remain in situ for six hours. A baseline blood sample will be taken prior to consuming one of four test meals. Further blood samples (5ml each time) after the ingestion of the test meals and collected into appropriate tubes for the preparation of serum. Serum iron concentrations will be measured in each sample using a colorimetric assay.
In this single blinded, randomised crossover human intervention study involving 16 volunteers, we aim to investigate if the protective/enhancing effect of alginate on iron absorption seen in vitro will also occur in vivo. To investigate it 16 volunteers will consume 2 meals on 2 different occasions. In addition we will also investigate whether the addition of iron inhibitor calcium will influence iron bioavailability. This part will be undertaken half of participants and will involve consuming further 2 meals on additional 2 occasions.
Primary objective: *To investigate whether alginate beads will provide protection for iron during the digestion process resulting in the delivery of increased amounts of bioavailable iron to the duodenum.*
Secondary objective*: To investigate whether the addition of calcium (inhibitor of iron absorption) influences iron bioavailability from iron-containing alginate beads. Half of the volunteers will participate in this part of the study.*

11. Methodology, Procedure and Analysis:

This study is a single blinded, randomized trial, with each participant acting as his/her own control, to measure the absorption of iron (20mg) as ferrous gluconate incorporated into alginate beads administered, compared with iron capsule (20mg also as ferrous gluconate) without alginate protection. All 16 participants will undertake this part of the study.
A further part of the study will investigate the modulatory effects of alginate on the inhibitory effect of calcium on iron absorption. It will involve consuming the iron (20mg) incorporated into the alginate beads with the addition of calcium capsules in the form of calcium phosphate (600mg) compared with an iron capsule (also 20mg as ferrous gluconate) without alginate protection followed by calcium capsules in the form of calcium phosphate (600mg). The addition of calcium will determine whether the alginate barrier is effective at reducing or preventing the inhibitory effect of calcium on iron absorption. Eight participants will undergo those 2 additional study days.
Participants will be randomly assigned to group A (n=8) or B (n=8). Group A will undergo 4 treatments whereas group B will undergo 2 treatments. In each case an oral dose of iron (approximately 20mg) will be given to the volunteers and we will investigate if there are differences in iron absorption.
The four tests will be as follows:
Test meal 1: for all volunteers (group A and B): 200ml cola jelly prepared from 200ml cola (Coca-Cola),10ml of commercially available Diet Cola flavoured Drink Concentrate (Sodastream, Lakeland) and gelatine (from animal source, Dr. Oetker, UK) with the addition of ferrous gluconate (approx 20mg +/- 2mg) in alginate beads (approx 22.2g) followed by 200ml of diet cola drink (Coca Cola) with 3 placebo capsules (filled with 50mg commercially available dextrose powder, 'Glucose Dextrose Powder', PharmacyNearU.com) acting as placebos for calcium and an iron capsules.)
Test meal 2: for 8 volunteers only (group A): 200ml cola jelly prepared from 200ml cola (Coca-Cola), 10ml of commercially available Diet Cola flavoured Drink Concentrate (Sodastream, Lakeland) and gelatine (from animal source, Dr. Oetker) with the addition of ferrous gluconate in alginate beads(approx.22.2g) followed by 200ml of a diet cola drink (Coca Cola) with 2 commercially available capsule containing calcium phosphate, (Freeda vitamins, USA) (300mg of calcium in each capsule, in total 600mg) and one placebo capsule in place of the iron capsule.
Test meal 3: for 8 volunteers only (group A): 200ml cola jelly prepared as above without iron containing alginate beads,2 calcium capsules (as above) and 1 ferrous gluconate capsule (prepared exclusively for each volunteer to match the iron content in the alginate beads administered in test meal 2) followed by 200ml diet cola drink.
Test meal 4 for all volunteers (group A and B): 200ml cola jelly prepared as above, 1 ferrous gluconate capsule (prepared exclusively for each volunteer to match the iron content in the alginate beads administered in test meal 1) followed by two dextrose (50mg each capsule, in total 100mg) placebo capsules in place of the calcium capsules and 200ml of diet cola drink.

Bioavailability will be measured by the post absorptive appearance and disappearance of iron in the serum following the test treatments. This is a standard procedure for generating absorption data. The use of placebo tablets (capsules containing dextrose) is necessary in order for the study to be single blinded.

The development of the iron-containing beads is novel on-going research. Therefore preparation of the iron-containing alginate beads imposes following limitations on the proposed study design: iron incorporation into the alginate beads is within 15% of that predicted; analysis of the iron (atomic absorption spectroscopy) incorporated

into the alginate beads is a three day procedure; long term storage method for the beads has not yet been established. As a consequence, iron containing alginate beads will be prepared for each volunteer approx. 48 hours before the test meal and stored in a refrigerator in a food grade kitchen (Institute of Food Research) at 4ºC. Sub-samples of each batch of beads administered to each volunteer will be collected and their iron content analysed using atomic absorption spectroscopy. Once the exact content of iron present in the beads administered to each specific volunteer in Test meal 1 and 2 is known (and recorded), exactly the same amount of ferrous gluconate will be weighed into a capsule and administered to that volunteer in a subsequent test meal (Test meal 4 and 3 respectively) after a minimum 7 day wash-out period. Due to above procedures scientists will not be blinded to the treatments order.

Participants will be recruited from the Norwich Research Park (NRP) and the surrounding Norwich area. Advertisements will be placed on the University of East Anglia (UEA) website, NRP newsletters, distributed via email to UEA staff and students (Annex 2), and placed on notice boards at IFR, the John Innes Centre (JIC), TGAC, UEA schools, local supermarkets, and other suitable locations (e.g. gyms) inviting anyone interested in receiving information about the study to contact named researchers by telephone or e-mail.

Potential volunteers who have expressed an interest in taking part will be sent a letter with the study advert and participant information sheet detailing the study.

Visit 1- Those volunteers responding positively after reading the information sheet will be invited to attend the Clinical Research and Trials Unit (CRTU) for an informal discussion about the study. This may last up to an hour. At the end of the discussion volunteers will be given a minimum of 72 hours to decide whether or not they wish to take part in the study.

Visit 2- Those volunteers who decide to take part in the study will attend the CRTU after an overnight fast for an eligibility assessment. Prior to the assessment the volunteers will be required to sign a consent form agreeing to take part in the study. During this visit the study scientist will also provide instructions on keeping a 1-day food diary to record the particpant's "pre-test diet" in preparation for the first experimental day (Visit 3). Volunteers will be asked to repeat the same pre-test diet prior each test visit. After consent is obtained the CRTU will complete a basic health questionnaire, measure height, weight, BMI and blood pressure. A 10ml of blood will be obtained to perform screening (full blood count, fasting glucose and ferritin) following which the volunteer will be offered a meal from 'packed lunch' Catering Direct menu.

Visit 3- On the day prior to the first test day, after recording all food and drink consumed during the day (pre-test diet) in a food diary. In cases where any food or drink was consumed in addition to the pre-test diet or was forgotten to be eaten, volunteers will be asked to note it down in the Record of Differences Diary. The volunteers will undergo an overnight fast from 10pm and attend the CRTU at approximately 8:00am the following morning. This visit as well as following one or three will take approximately 6 hours. Prior to cannulation a CRTU nurse will take and record the volunteer's blood pressure. If the volunteer's blood pressure is <90/50 or <95/55 if symptomatic or >160/100 three further measurements may be taken and recorded at 5 minute intervals. If the blood pressure measurement remains outside these ranges after the third measurement, the volunteer will be excluded from the study, referred to their G.P. and the study day cancelled.

Female volunteers will be asked by a study scientist at the start of each experimental day if they are menstruating and if they are not, then they will be asked how many days ago menstruation has completed. This information may help to explain potential fluctuations in ferritin levels and potential differences in iron appearance in the serum during different experimental days. Menstrual iron losses may affect biomarkers of iron status such as ferritin which may in turn affect iron absorption.
If volunteers' blood pressure is within the acceptable range a cannula will be inserted and the baseline blood sample will be taken, followed by administration of the test treatment. Subsequent blood samples will be collected at 20, 40, 60, 80, 100, 120, 150, 180, 240, 300 and 360 min after the ingestion of the oral doses of iron. A meal from 'packed lunch' Catering Direct menu will be provided after the final 6 hour blood sample.

Visits 4, 5 and 6- the remaining one or three test phases will each be separated by a minimum of one week. Each of the test days will be identical to visit 3 except for the test treatment consumed. In summary there are 4 or 6 visits in total for the volunteer depending to which group he/she will be randomised. It is anticipated that volunteers will participate for approximately 16 weeks but this is dependent upon each individual's own personal commitments.

We will measure the concentration of iron in the serum at the time points described above and calculate the maximum concentrations reached, the time at which the maximum concentration was reached and the area under the curve values for each volunteer. Statistical analysis will be undertaken to compare values obtained from the different test treatments.
In addition we will measure ferritin and serum transferrin receptor (using elisa assays) in baseline blood samples from every experimental day to calculate body iron in order to fully characterise the iron status of the study group and to investigate the reliability and fluctuation in body iron measurements.
Furthermore CRP assessment from baseline blood samples will be performed on each experimental day to establish if serum iron appearance could be affected by inflammation. This analysis will be performed at an accredited pathology laboratory at the NNUH, Norwich.

12. Resources required:

All clinical procedures (venepuncture, cannulation, consumption of test meal) most non clinical procedures (initial interview, consent signing) will be performed at the Clinical Research and Trials Unit (CRTU). The one day food diary will be completed by the volunteers at home or elsewhere. Analysis of the samples will be performed at University of East Anglia.

The amount of payment to the CRTU has been agreed and it will be transferred as a lump sum including all procedures, nurse time etc, as listed above, which will be performed at CRTU.

All the analysis will be performed at UEA by the PhD student.

13. Source of Funding

This project is in collaboration with IFR and is part of a doctoral training award.

The grant is secured and has been funded by Biotechnology & Biological Sciences Research Council (BBSRC) Diet and Health Research Industry Club (DRINC).

14. Has this project been peer reviewed? Please could you include details of who the project has been peer reviewed by.

This study has been peer reviewed by Prof. Dr.med. Michael B. Zimmermann and Dr Ines Egli, Institute of Food, Nutrition and Health, ETH Zurich. The letter with comments regarding the scientific justification of the research, study design and methods is attached to this application.

15. Ethical issues (Please also complete research safety checklist even if no risks are identified)

- *Volunteer exclusion criteria- These criteria have been selected to ensure that the scientific data collected during the study will be robust and confounding factors minimised. There are also exclusion criteria which are necessary for the wellbeing of the volunteers. For example, the following types of volunteers will be excluded from participation: pregnant woman due to their increased iron demands, those with type-1 or type-2 diabetes because the study involves fasting on several occasions; people with decreased or elevated blood pressure; volunteers related to or living with a member of the study team in order to avoid the possibility of undue pressure being placed on either the researcher or potential volunteers. In addition factors that may influence the study results have also been considered, For example: medications, nutritional supplements, high (may see too small effect on iron absorption) or too low iron stores (risk of anaemia for volunteers).*

- *Venepuncture and cannulation- there is a risk of minor discomfort and bruising to the volunteers during the phlebotomy procedure. Nurses performing venepuncture are experienced in this procedure thereby minimising the risk. The total volume of blood for this study is 130 ml (if volunteer will undergo 2 test days) or 250ml (if volunteer will undergo 4 test days)which will be collected on up to 5 different occasions (10ml during screening and 60ml for each of 2 or 4 visits). This will be collected over 10-16 week period. To eliminate a small risk of becoming anaemic, volunteers with too low iron status during the screening will be excluded from the study.*

- *Volunteer fasting- Blood samples for this study will be collected over a 6 hour time period following an overnight fast which will be approximately 16 hours of fasting in total. Because the volunteers are fasted for such a long time they will be asked to attend CRTU early in the morning. A light meal will be given afterwards. Volunteers will be advised to drink water during the fasting period.*

- *Flagged blood results- As a part of screening procedure all samples will be sent to the Norfolk and Norwich University Hospital Pathology Department for full blood count ferritin and glucose analysis. It will be possible that some of these results will be outside the standard reference ranges. Sample results will be first reviewed by CRTU nurse and if any of the results will be flagged they will be forwarded to study Medical Advisor who will advise on appropriate course of action. Volunteers who will have flagged results may be advised to*

contact their GP for follow up. All blood results whether flagged or not will be forwarded to the volunteers GP.

- *Confidentiality- All personal data collected from volunteers will be kept in locked filing cabinets at UEA. Once recruited onto the study volunteers will be assigned a unique code number. This number will be used to label all blood samples and data arising from them. All coded data will be kept separately from personal data.*

- *Informed consent- to make an informed decision whether to take part in the study, the volunteers will have received an information sheet and attended one to one pre study talk/meeting. Consent will be obtained by PhD student who will be overseen by a supervisor experienced in conducting human intervention studies. Supervision will be continued up to the point where supervisor is confident with students' abilities to continue on their own.*

- *Researcher/volunteer bias- The volunteers will be randomized to group A or B. Group A will undergo 4 study days and group B will undergo 2 study days. In addition the volunteers will not know which test product they will receive on each occasion. However due to the design of the research (the need of preparation of alginate beads prior each experimental day) this study will be single blinded to ensure that the iron containing alginate beads are prepared in an appropriate way for each test meal.*

- *The number and timings of blood samples on each of the two/four test days has been selected based on the previous experience of the chief investigator measuring iron absorption from test meals, and supporting data from the literature. The rate of iron absorption depends on an individual. Nonetheless peak serum concentrations are usually reached within 150 to 180 minutes and return to the base line within 6 hours. In order to undertake appropriate statistical calculations it is crucial for the absorption curve to be well characterised and consequently the timings of blood sample collections have been chosen to reflect this requirement.*

16. Proposed start and finish dates:

Start date: *01.01.2012* **Finish date:** *01.11.2012*

17. Where will the research be carried out?

All clinical procedures will be performed by experienced nurses at Clinical Research and Trials Unit (CRTU). Analysis of the samples collected as well as data analysis will be performed at University of East Anglia, in BMRC building. All non clinical procedures involving volunteers, processing and analysis of obtained blood samples of this study will be performed by Principal Investigator for UEA Anna Wawer. For better participants understanding on all study paperwork PI for UEA Anna Wawer is referred as study manager.

18. Do you need to survey UEA students or staff outside the Faculty of Health? *NO* If so, you need to get approval in principle from

the Dean of Students prior to applying to the FOH Ethics Committee (see hyperlink below). Please attach a copy of approval in principle to this application form.

https://www.uea.ac.uk/polopoly_fs/1.151266!survey_form.pdf

19. Information sheets and consent forms must be appended (see the NRES site for models - www.nres.npsa.nhs.uk).
NB The Committee request that you do not produce your Participant Information Sheet in two parts (to avoid duplication); and that you ensure that participants are required to initial the boxes on your consent forms.

20. Checklist

Have you completed all sections of the application in language which will be understood by lay people? ☐

Has your supervisor signed the form? ☐

Have you included your academic address (not your home address)? ☐

Have you numbered all the pages in your protocol/attachments? (If the pages are not numbered the Committee may return your application) ☐

Have you included the following documents, if applicable?

Protocol ☐

Gatekeeper consent ☐

Consent forms ☐

Participant information sheets (using NRES format) ☐

Letters to participants ☐

Copies of questionnaires ☐

Copies of correspondence from other ethic committees ☐

Research Safety Checklist ☐

Dean of Student Office approval in principle for survey ☐

Have you proof-read your application to check for typographical and grammatical errors? ☐

Have you included a header and footer on each page with your name, date of submission and page number? ☐

Have you included 16 photocopies? ☐

Have you e-mailed a copy to the Faculty Research Office? ☐

Supervisory arrangements for **STUDENT PROJECTS ONLY**

Degree/Course PhD..

School MED…………………………………………………. ….

Academic Supervisor: Professor Susan Fairweather-Tait

I have read this application and can confirm that I am taking supervisory responsibility for this project.

In the case of a student research outside the normal course requirements I confirm that I am happy to take responsibility for the quality of protocol design, the provision of necessary resources, statistical support and usual supervision and governance of the student.

Project Supervisor's signature Date

28th Sept 2011………………

Post Held Professor of mineral metabolism
