# Supplementary material for: Alginate Inhibits Iron Absorption from Ferrous Gluconate in a Randomized Controlled Trial and Reduces Iron Uptake into Caco-2 Cells
Source: PLoS One. 2014 Nov 12;9(11):e112144. doi: 10.1371/journal.pone.0112144 (PMC4229116; doi:10.1371/journal.pone.0112144)
Supplement: Form S6 — Ethics approval letter. (PDF) [file pone.0112144.s011.pdf]

Faculty of Medicine and Health Sciences Research Ethics Committee

University of East Anglia

Anna Wawer
Department of Nutrition
BMRC Building
Norwich Medical School
University of East Anglia
Norwich
Norfolk
NR4 7TJ

21st November 2011

Research & Enterprise Services
REN West (SCI)
University of East Anglia
Norwich
NR4 7TJ

Email: fmh.ethics@uea.ac.uk
Direct Dial: +44 (0) 1603 59 157197

Web: http://www.uea.ac.uk

Dear Anna

Project title: Study to measure the absorption of iron from ferrous gluconate incorporated into alginate beads – Reference: 2011/2012-05

The resubmission of your above proposal has been considered by the Chair of the Faculty Research Ethics Committee and we can confirm that your proposal has been approved.

Please could you ensure that any amendments to either the protocol or documents submitted are notified to us in advance and also that any adverse events which occur during your project are reported to the Committee. Please could you also arrange to send us a report once your project is completed.

The Committee would like to wish you good luck with your project

Yours sincerely

Yvonne Kirkham

Yvonne Kirkham
Project Officer
